# Mycobiota Associated with the Vascular Wilt of Poplar

**DOI:** 10.3390/plants10050892

**Published:** 2021-04-28

**Authors:** Hanna Kwaśna, Wojciech Szewczyk, Marlena Baranowska, Ewa Gallas, Milena Wiśniewska, Jolanta Behnke-Borowczyk

**Affiliations:** 1 Department of Forest Pathology, Poznań University of Life Sciences, Wojska Polskiego 71c, 60-625 Poznań, Poland; wojciech.Szewczyk@up.poznan.pl (W.S.); ewa.gallas@up.poznan.pl (E.G.); milena.wisniewska@up.poznan.pl (M.W.); jolanta.behnke@up.poznan.pl (J.B.-B.); 2 Department of Silviculture, Poznań University of Life Sciences, Wojska Polskiego 71a, 60-625 Poznań, Poland; marlena.baranowska@up.poznan.pl

**Keywords:** fungi, pathogens, plantation, poplar hybrids, vascular wilt

## Abstract

In 2017, a 560-ha area of hybrid poplar plantation in northern Poland showed symptoms of tree decline. The leaves appeared smaller, yellow-brown, and were shed prematurely. Twigs and smaller branches died without distinct cankers. Trunks decayed from the base. The phloem and xylem showed brown necrosis. Ten percent of the trees died 1–2 months after the first appearance of the symptoms. None of these symptoms were typical for known poplar diseases. The trees’ mycobiota were analysed using Illumina sequencing. A total of 69 467 and 70 218 operational taxonomic units (OTUs) were obtained from the soil and wood. Blastocladiomycota and Chytridiomycota occurred only in the soil, with very low frequencies (0.005% and 0.008%). Two taxa of Glomeromycota, with frequencies of 0.001%, occurred in the wood. In the soil and wood, the frequencies of Zygomycota were 3.631% and 0.006%, the frequencies of Ascomycota were 45.299% and 68.697%, and the frequencies of Basidiomycota were 4.119% and 2.076%. At least 400 taxa of fungi were present. The identifiable Zygomycota, Ascomycota, and Basidiomycota were represented by at least 18, 263 and 81 taxa, respectively. Many fungi were common to the soil and wood, but 160 taxa occurred only in soil and 73 occurred only in wood. The root pathogens included species of Oomycota. The vascular and parenchymal pathogens included species of Ascomycota and of Basidiomycota. The initial endophytic character of the fungi is emphasized. Soil, and possibly planting material, may be the sources of the pathogen inoculum, and climate warming is likely to be a predisposing factor. A water deficit may increase the trees’ susceptibility. The epidemiology of poplar vascular wilt reminds grapevine trunk diseases (GTD), including esca, black foot disease and Petri disease.

## 1. Introduction

*Populus* is a genus of deciduous trees in the family Salicaceae, native to most of the Northern Hemisphere. They are among the fastest-growing trees, and the most efficient in terms of sustainability. Poplar is significant because of: (i) its rapid production of wood (in Europe, 1 m^3^ of lumber can be produced on average in 15 years, six times faster than with oak); (ii) its very versatile wood, with an excellent ratio between specific weight and mechanical features, making it suitable for furniture, plywood and the paper industry; (iii) its excellent capacity for purifying the air by capturing CO_2_ and storing it in the biomass (1 ha can capture 11 t CO_2_/year); (iv) its capacity for purifying water while acting as a green filter, absorbing nitrates and sediments; (v) its potential for biofuel production using the coppicing method; (vi) the possibility for its cultivation on abandoned and degraded land, thus optimizing land use.

Poplar is an important source of wood for pulp and paper products, but mostly paper, for which worldwide production reaches 420 Mt, including 5 Mt in Poland [1]. Its wood is also suitable for use as a renewable energy source. The development of renewable sources for energy purposes has been substantially supported and promoted by a European Union Directive. Poland is obliged to obtain at least 30% of its energy from renewable sources by 2030 (Directive (EU) 2018/2001). Wood that is suitable for renewable energy includes that derived from trees grown in short- and medium-rotation plantations, often on agricultural land or non-forested areas. Plantations based on varieties of *Acacia* and *Eucalyptus* have been particularly effective in tropical countries with favourable climate and soil conditions for faster growth; *Eucalyptus* has produced 25 m^3^ of wood per ha annually, compared with 7–8 m^3^ in the temperate climate zone (1). Plantations of fast-growing trees are now also being established in the temperate zone. The most promising genus in Poland is poplar (*Populus* spp.), with plantations usually in short- (up to 10 years) or medium-rotation (up to 15–25 years) coppice systems [2,3,4].

Hybrid poplar trees are often the progeny of crosses between cottonwood (*Populus deltoides* W. Bartram ex Marshall) and black poplar (*Populus nigra* L. ‘Italica’). They have the advantages of: (i) rapid growth (1.5–2.5 m per year), (ii) a large range of hardiness zones (3–9), (iii) high productivity resulting from a prolonged vegetation period, and (iv) better resistance to pests and diseases [5].

Poplars are frequently attacked by microorganisms that cause discolorations, necrosis, depressions, deformations (thickening of the trunk and branches, the abnormal proliferation of the underlying phloem, the formation of the corky ridges or woody galls). Stresses predispose trees to infection by phytopathogens. Attacks on the trunk and branches of younger trees often kill the main shoot.

The bark necrosis of poplars can be caused by *Discosporium populeum* (Sacc.) B. Sutton (=*Chondroplea populea* (Sacc.) Kleb. = *Dothichiza populea* Sacc. Sacc. & Briard, anamorph of *Cryptodiaporthe populea* (Sacc.) Butin). Necrosis and cankers are often caused by *Cytospora* spp. (*C. populina* (Pers.) Rabenh. = *C. ambiens* Sacc., teleomorph *Valsa ambiens* (Pers.) Fr., and *C. nivea* Fuckel, teleomorph *V. nivea* (Hoffm.) Fr.). Cankers can be caused by *Entoleuca mammata* (Wahlenb.) Rogers and Ju (=*Hypoxylon mammatum* (Wahl.: Fr.) Karst.). Sooty-bark canker is caused by *Sclerencoelia pruinosa* (Ellis and Everh.) Pärtel and Baral (=*Encoelia pruinosa* (Ell. and Ev.) Torkelsen and Eckblad). Black or target canker can be caused by *Ceratocystis fimbriata* Ellis and Halst. Other agents of necrosis and cankers or wood rots and bark alterations, of which the incidence is more local and/or secondary, include *Boeremia populi* (Gruyter and Scheer) Jayawardena, Jayasiri and Hyde (=*Phoma exigua* var. *populi* Gruyter and Scheer), *Botryodiplodia populea* Zhong, *Diplodia tumefaciens* (Shear) Zalasky (the anamorph of *Keissleriella emergens* (Karst.) Bose), *Fusarium* spp., *Neofusicoccum ribis* (Slippers, Crous and M.J. Wingf.) Crous, Slippers and Phillips (=*Dothiorella gregaria* Sacc., the anamorph of *Botryosphaeria dothidea* (Moug.) Ces. and De Not), *Neonectria ditissima* (Tul. and C. Tul.) Samuels and Rossman (with anamorph *Cylindrocarpon mali* (Allesch.) Wollenw.), *Phomopsis* spp., *Rhytidiella moriformis* Zalasky, *Rhytidiella baranyayi* Funk and Zalasky, and basidiomycetous *Erythricium salmonicolor* (Berk. and Broome) Burds. (=*Corticium salmonicolor* Berk. and Broome). Damage to heartwood can be caused by bacteria (*Erwinia nimipressuralis*). Disease of the leaves are usually caused by *Melampsora medusae* Thüm. (rust), *Venturia tremulae* Aderh. (scab, shoot blight), *Sphaerulina musiva* (Peck) Quaedvl., Verkley and Crous (=*Septoria musiva* Peck), and *Marssonina* spp. Most infections of woody tissues are initiated by wind-borne ascospores, which are forcibly ejected from perithecia during periods of damp weather. Fungi infect trees through wounds and invade the inner bark and cambium.

In 2017, a 560 ha plantation of hybrid poplar (*P. deltoides* × *P. nigra*) in northern Poland showed symptoms of tree decline. The leaves of the diseased trees appeared smaller, turned yellow-brown, and were shed prematurely. Twigs and smaller branches died without definite cankers. The bark of the entire trunk was sunken and discolored, often loosened and split. It often fell off, exposing wet wood. The trunks decayed from the base. The phloem showed brown necrosis. Ten percent of the trees died in 1–2 months (in June) after the first appearance of the symptoms. None of the observed symptoms were typical for known poplar diseases.

The objectives of the study on the structure of the fungal communities present in the rotten wood of poplar trunks and in the soil were to: (i) determine the abundance and diversity of pathogens and other fungi; (ii) identify interactions among fungi that may contribute to the disease progress; (iii) assess associations between the disease and global warming, with consequences for host and pathogen physiology, reproduction, survival, spatial and temporal distribution, resource availability and competition.

## 2. Materials and Metods

### 2.1. Site and Sampling

**Figure 1:**
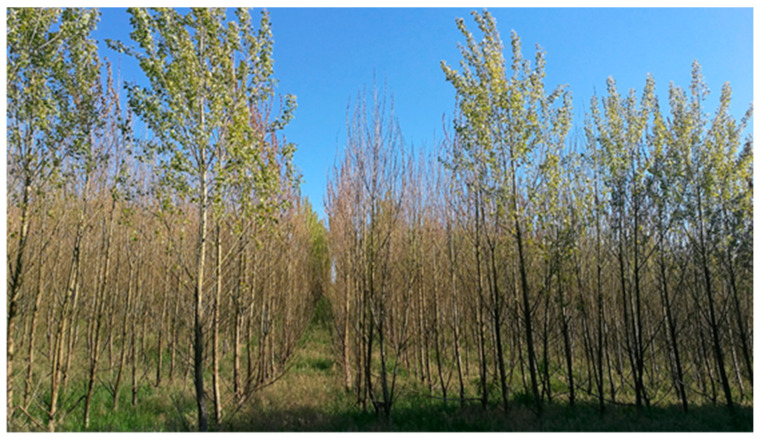
Poplar plantation with diseased trees.

**Figure 2:**
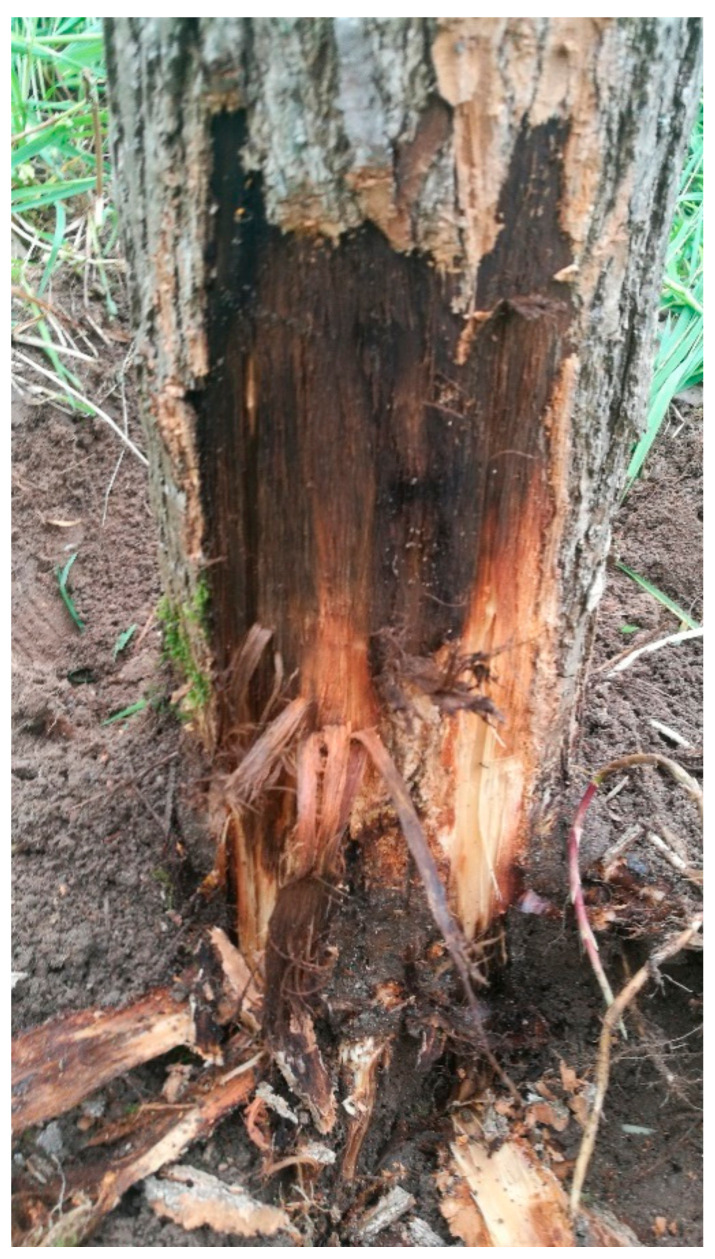
Necrosis and decay at the base of the trunk of a diseased poplar.

The study was carried out in the Łoża, Czarne District, Człuchów County, Pomeranian Voivodeship, northern Poland (53°41′29″ N 17°04′19″ E), in a 560 ha plantation of 5–6-year-old hybrid poplar (*P. deltoides* × *P. nigra*, cultivar AF2, from Italy) showing symptoms of crown decline, trunk-base decay (520 ha) and tree death (40 ha) (Figure 1 and Figure 2). The plantation was so intensively affected that the inclusion of a control (healthy plantation) from the same area with the same conditions of climate and soil was impossible.

The trees were grown at a density of 425 trees/ha (4 m × 4m spacing), and had a mean diameter of 9–10 cm at breast height. The post-agricultural soil was sandy loam, consisting of sand (60%), silt (20%) and clay (20%), with a low humus level. The former crop was rye (*Secale cereale* L.). The average temperature is 7.9 °C and the rainfall is 680 mm.

The understorey vegetation included *Achillea millefolium* L., *Agrostis stolonifera* L., *Artemisia absinthium* L., *Artemisia vulgaris* L., *Cichorium intybus* L., *Elymus repens* (L.) Gould, *Lamium purpureum* L., *Lolium perenne* L., *Papaver rhoeas* L., *Poa annua* L., *Poa pratensis* L., *Poa trivialis* L., *Polygonum aviculare* L., *Polypodium vulgare* L., *Polytrichum commune* Hedw., *Stellaria media* Hist. Pl. Dauphiné, *Taraxacum officinale* F.H. Wigg., and *Trifolium arvense* L.

Five wood cores; 10 cm long and 3 cm in diam., each including bark, phloem and xylem, were sampled from the bases of the necrotic trunks of five symptomatic trees, 0 cm and 50 cm above the ground, with a Pressler borer. The core samples were surface-sterilized and ground to sawdust with a cordless SPARKY BUR2 15E drill. Additionally, five subsamples of soil were taken as cylindrical cores, 10 cm long and 5 cm in diam., from the surroundings of roots of five symptomatic trees. They were placed in sterile glass containers and refrigerated for 48 h.

### 2.2. DNA Extraction, Amplification and Illumina Sequencing

Five samples of sawdust were prepared from five wood cores in the SPEXTM SamplePrepTM Freezer/MillTM cryogenic mill. The wood’s genomic DNA was extracted from each of five 30 mg heavy sawdust samples using a Plant Genomic DNA Purification Kit (Thermo Scientific, Carlsbad, California, USA). The soil’s genomic DNA was extracted from each 300 mg soil subsample using a Power SoilM DNA Isolation Kit (MO BIO Laboratories, Carlsbad, CA, USA).

The rDNA was amplified with fungi specific primers ITS1 FI2 (5′-GAACCWGCGGARGGATCA-3′) [6] and 5.8 S (5′-CGCTGCGTT CTTCATCG-3′) [7].

The PCR reaction mixture consisted of 12.5 μL of 2 × Mix PCR (A & A Biotechnology, Gdańsk, Poland), 0.2 μM of each primer, 1.5 μL purified and diluted DNA, and 10.6 μL water. The DNA amplification was performed under the following conditions: denaturation at 94 °C for 5 min followed by 35 cycles of denaturation at 94 °C for 30 s, annealing at 56 °C for 30 s, elongation at 72 °C for 30 s, and a final elongation at 72 °C for 7 min. The visualization of 5-μL amplicons was performed in 1.0% agarose gel dyed with Midori Green Advance DNA (Genetics). The pooled PCR products were purified using a MinElute PCR Purification Kit (Qiagen, Hilden, Germany). The concentration of PCR products was quantified using a Qubit 2.0 Fluorometer (Life Technologies, Carlsbad, CA, USA), and an equimolar mix of PCR products from each sample was prepared. The amplicons were sequenced using the Illumina system in the Genomic Laboratory, DNA Research Center, Rubież 46, Poznań, Poland.

### 2.3. Bioinformatics Analysis

A table of Operational Taxonomic Units (OTUs) was prepared by PIPITS, version 1.2.0 [8]. The read-pairs were joined with PEAR, version 0.9.6 [9], filtered with a quality threshold of q = 30 by FASTX-toolkit, version 0.0.13 (http:hannonlab.cshl.edu/fastx_toolkit/index.html, accessed on 26 April 2012) converted to the Fasta format, and merged into a single file. The prepared sequences were de-replicated, and subregions of ITS were selected with the use of ITSx, version 1.0.11 [10]. Unique sequences and those shorter than 100 bp were removed. The remaining sequences were clustered with 97% sequence identity. The resulting representative sequences for each cluster were subjected to chimera detection and removal using the UNITE UCHIME reference dataset, version 6.0 (https://unite.ut.ee/index.php (accessed on 26 April 2012)). The input sequences were then mapped onto the representative sequences, and taxonomy was assigned using RDP Classifier, version 2.10.2 [11] against the UNITE fungal ITS reference database, version 11.2 [12]. This process resulted in the creation of a table of OTUs. The sequences were identified by comparison with reference sequences from the National Center for Biotechnology Information (NCBI) database.

The abundance of fungi was defined as the average number of OTUs from five subsamples. The frequency of an individual taxon was defined as the percentage (%) of OTUs in the total number of OTUs. The similarity and relationships between the fungal communities from the soil and wood is shown by a heat map.

### 2.4. Statistical Analyses

The differences in the abundance of microfungi in the soil and wood were analysed with chi-squared tests (*χ*^2^). The diversity between the communities of microfungi was compared with Margalef’s diversity index (*D*_Mg_), Shannon’s diversity index (*H*), Simpson’s diversity index (*D*), Shannon’s evenness index (*E*) and Berger–Parker’s index (*d*) [13].

## 3. Results

**Figure 3:**
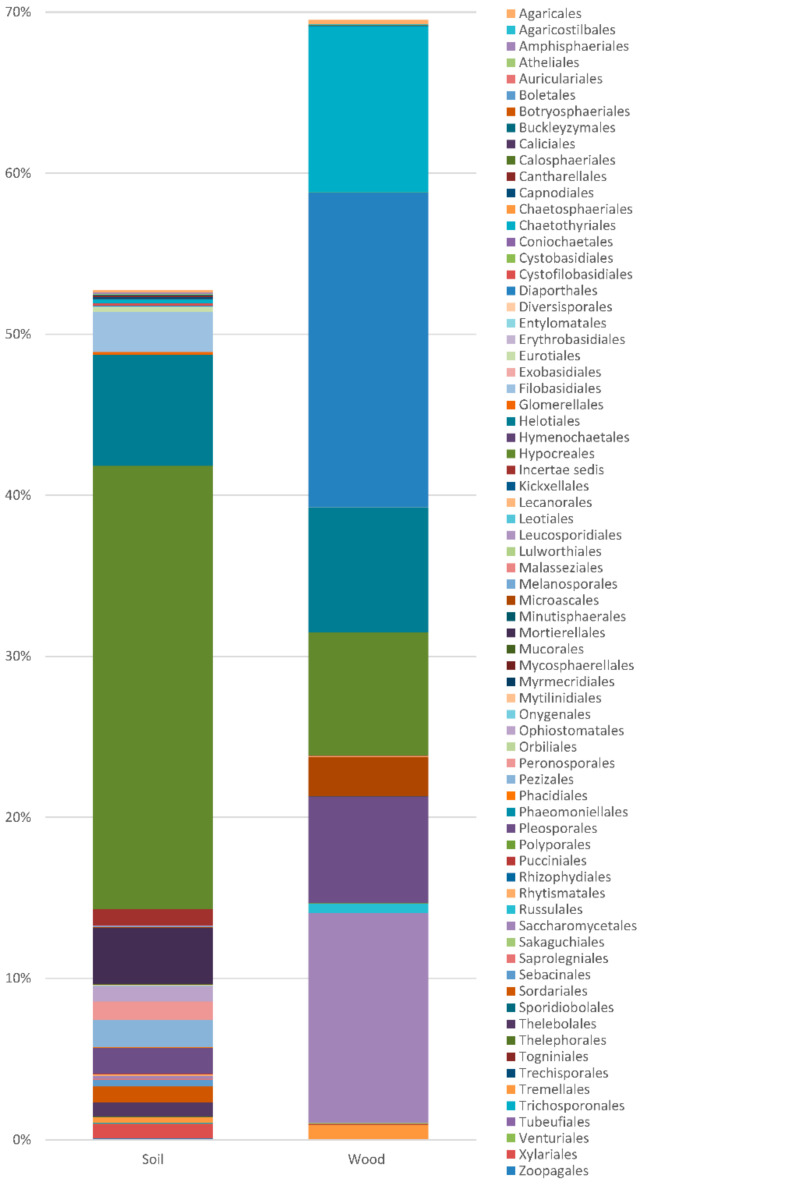
Frequency of the fungi in taxonomic orders.

Totals of 69 467 and 70 218 OTUs were obtained, respectively, from the soil and wood of the *Populus* hybrid using the Illumina sequencing technique (Table 1, Figure 3). Of these, 44 506 (64%) and 53 592 (76%) were of fungi known from culture, and 24 961 (36%) and 16,628 (24%) were unidentified fungi and other organisms. Fungi from Blastocladiomycota, Chytridiomycota, Glomeromycota, Zygomycota, Ascomycota and Basidiomycota were detected. Blastocladiomycota and Chytridiomycota occurred only in the soil, with very low frequencies of 0.005% and 0.008%. Two taxa of Glomeromycota with a frequency of 0.001% occurred in the wood. The frequencies of Zygomycota in the soil and wood were 3.631% and 0.006%, the frequencies of Ascomycota were 45.299% and 68.697%, and the frequencies of Basidiomycota were 4.119% and 2.076%. The samples were colonized by at least 400 taxa of fungi. Identifiable Zygomycota, Ascomycota, and Basidiomycota were represented by at least 18, 263 and 81 taxa, respectively. Many fungi were common to the soil and wood, but 160 taxa occurred only in the soil, and 73 occurred only in the wood.

**Figure 4:**
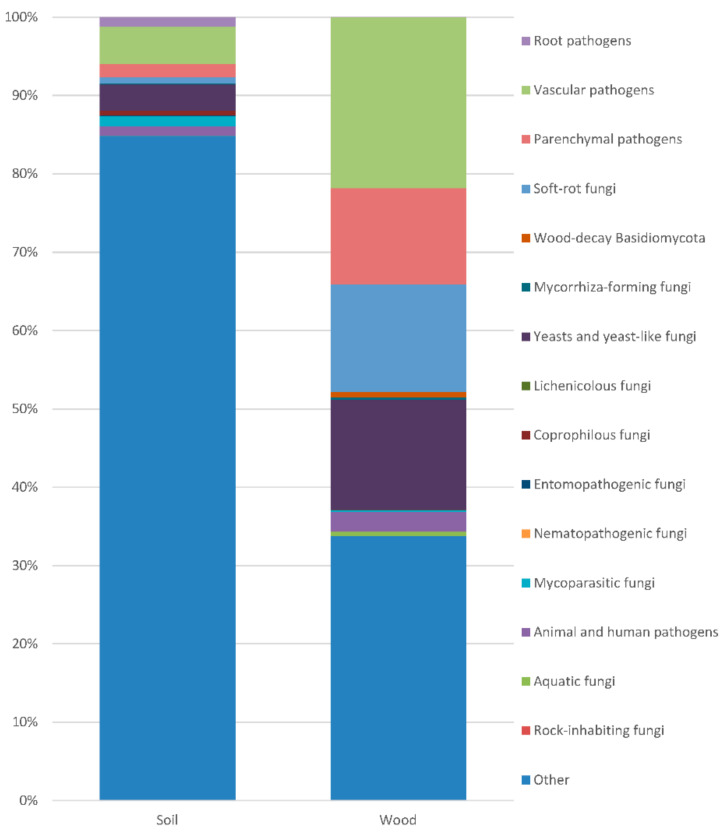
Frequency of the fungi in specific trophic groups.

Saprotrophs were the most abundant (Figure 4). In the soil, their frequency exceeded 80%. In the soil, the most common (with frequency > 0.1%) were species of *Mortierella* (Zygomycota), *Alatospora*, *Clonostachys*, *Dendryphion*, *Emericellopsis*, *Exophiala*, *Halenospora*, *Lambertella*, *Leptodontidium*, *Magnohelicospora*, *Metarhizium*, *Neobulgaria*, *Nigrospora*, *Penicillium*, *Petriella*, *Pleotrichocladium*, *Pseudeurotium*, *Tetracladium*, *Tricharina* and *Trichoderma* (Ascomycota), *Coprinellus*, *Cryptococcus*, *Fibulobasidium*, *Phaeotremella* and *Solicoccozyma* (Basidiomycota).

Individual taxa of obligate or facultative phytopathogens were more or less frequent.

The root pathogens included species of *Aphanomyces*, *Globisporangium*, *Phytophthora* and *Pythium* (Oomycota: 1.17%), and *Truncatella* (Ascomycota: 0.003% in the soil, 0. 001% in the wood).

Vascular pathogens included species of *Cadophora*, *Dactylonectria*, *Debaryomyces*, *Fusarium*, *Fusicolla*, *Graphium*, *Hymenoscyphus*, *Ilyonectria*, *Microdochium*, *Neonectria*, *Ophiostomataceae*, *Phaeoacremonium*, *Phaeomoniella*, *Phialophora*, *Sporothrix*, *Thelonectria* and *Verticillium* (Ascomycota: 4.783% in soil, 21.831% in the wood).

The parenchymal pathogens included species of *Alternaria*, *Boeremia*, *Cladosporium*, *Coniochaeta*, *Cosmospora*, *Cytospora*, *Diaporthe*, *Didymella*, *Epicoccum*, *Herpotrichia*, *Hypoxylon*, *Lophiostoma*, *Mycosphaerella*, *Neoascochyta*, *Neocatenulostroma*, *Neofabraea*, *Neoleptosphaeria*, *Neopyrenochaeta*, *Paraphoma*, *Phaeoisaria*, *Phaeosphaeria*, *Phaeosphaeriopsis*, *Phoma*, *Phomopsis*, *Plectosphaerella*, *Pseudocercospora*, *Pyrenochaeta*, *Pyrenochaetopsis*, *Scytalidium*, *Sphaeropsis*, *Stemphylium*, *Sydowia*, *Valsa*, *Volutella* and *Xenoramularia* (Ascomcota: 1.647% in the soil, 11.645% in the wood), and *Armillaria*, *Aurantiporus*, *Chondrostereum*, *Fomitopsis*, *Peniophora* and *Serpula* (Basidiomycota: 0.026% in the soil, 0.618% in the wood).

The soft-rot fungi included species of *Alatospora*, *Alternaria*, *Cadophora*, *Chaetomium*, *Cladosporium*, *Clonostachys*, *Exophiala*, *Halenospora*, *Leptodontidium*, *Neosetophoma*, *Orbilia*, *Phialophora*, *Plagiostoma*, *Sydowia* and *Tricladium* (Ascomycota: 0.821% in the soil, 13.757% in the wood).

The wood-decay Basidiomycota included the white rot fungi *Armillaria mellea*, *Aurantiporus fissilis*, *Bjerkandera adusta*, *Chondrostereum purpureum*, *Hyphodontia pallidula* and *Peniophora*, and the brown rot fungus *Fomitopsis piniola*. They occurred with frequencies of 0.028% in the soil and 0.62% in the wood.

The mycorrhiza-forming fungi present in the soil and wood included 12 taxa: arbuscular *Entrophospora* (Glomeromycota: 0.001% in the wood); ectomycorrhizal *Cenococcum geophilum* (Ascomycota; 0.039% in the soil), *Hymenogaster arenarius*, *Inocybe curvipes*, *Laccaria* sp., *Serendipita vermifera* and *Tomentella* (Basidiomycota: 0.048% in the soil, 0.019% in the wood); ectendomycorrhizal *Chloridium paucisporum* and *Leptodontidium* sp. (Ascomycota), and *Camarophyllus* sp., *Efibulobasidium* sp. and *Hebeloma mesophaeum* (Basidiomycota: 0.039% in the soil, 0.254% in the wood).

The yeasts and yeast-like fungi present in the soil and wood included 52 taxa: *Aureobasidium melanogenum*, *Blastobotrys* spp., *Candida* spp., *Capnobotryella renispora*, *Cladophialophora* spp., *Cyphellophora sessilis*, *Debaryomyces hansenii*, *Exophiala* spp., *Meyerozyma guilliermondii*, *Micarea agnata*, *Nakazawaea* spp., *Saccharomyces cerevisiae*, *Yamadazyma mexicana*, *Yarrowia lipolytica* and *Xanthoparmelia subchalybaeizans* (Ascomycota: 0.296% in the soil, 13.072% in the wood); *Apiotrichum dulcitum*, *Bensingtonia* spp., *Buckleyzyma aurantiaca*, *Bullera croce*, *Bulleromyces albus*, *Cryptococcus* spp., *Curvibasidium pallidicorallinum*, *Cystobasidium* spp., *Cystofilobasidium* spp., *Erythrobasidium hasegawianum*, *Fellomyces* spp., *Fellozyma inositophila*, *Fibulobasidium inconspicuum*, *Filobasidium wieringae*, *Hannaella zeae*, *Itersonilia perplexans*, *Kockovaella machilophila*, *Kondoa yuccicola*, *Kwoniella newhampshirensis*, *Malassezia* spp., *Mrakia frigida*, *Naganishia cerealis*, *Phaeotremella* spp., *Piskurozyma* sp., *Rhodotorula* spp., *Saitozyma podzolica*, *Sakaguchia lamellibrachiae*, *Sirotrema translucens*, *Slooffia pilatii*, *Solicoccozyma* spp., *Sporobolomyces* spp., *Symmetrospora coprosmae*, *Tausonia pullulans*, *Tremella encephala*, *Trichosporon otae* and *Vishniacozyma carnescens* (Basidiomycota: 3.061% in the soil, 1.017% in the wood).

The lichenicolous fungi present in the soil and wood included eight taxa: *Bacidina* sp., *Knufia peltigerae*, *Lecania cyrtella*, *Lepraria caesiella*, *Micarea agnata*, *Physcia tenella*, *Pilophorus strumaticusa* and *Xanthoparmelia subchalybaeizans* (Ascomycota: 0.02% in the soil, 0.068% in the wood).

The coprophilous fungi present in the soil and wood included 10 taxa: *Ascobolus* sp., *Cercophora* sp. *Coniochaeta* sp., *Lophotrichus* sp., *Meyerozyma guilliermondii*, *Petriella sordida*, *Phaeoisaria*, *Podospora appendiculata* (forest specific), *Preussia* spp. and *Schizothecium glutinans* (Ascomycota: 0.548% in the soil, 0.002% in the wood). The entomopathogenic fungi present in the soil and wood included three taxa: *Beauveria bassiana* and *Cordyceps* spp. (Ascomycota: 0.096% in the soil, 0.023% in the wood), and *Kwoniella* spp. (Basidiomycota: 0.016% in the soil, 0.003% in the wood).

The nematopathogenic fungi included one species, *Myzocytiopsis* sp. (Oomycota: 0.005% in the soil).

The mycoparasitic fungi present in the soil and wood included 18 taxa: *Syncephalis* sp. (Zygomycota: 0.107% in the soil), *Angustimassarina* spp., *Cladosporium* spp., *Clonostachys* spp., *Coniochaeta* sp., *Cordyceps* spp., *Cosmospora* sp., *Dissoconium eucalypti*, *Infundichalara microchona*, *Macroconia sphaeriae*, *Melanospora kurssanoviana*, *Nigrograna mycophila* and *Scytalidium lignicola* (Ascomycota: 1.063% in the soil, 0.056% in the wood), *Cystobasidium* spp., *Geotrichopsis mycoparasitica*, *Gymnopus androsaceus*, *Minimedusa polyspora* and *Phaeotremella frondosa* (Basidiomycota: 0.16% in the soil, 0.139% in the wood).

The animal and human pathogens included *Coniochaeta*, *Exophiala*, *Graphium* spp., *Lophotrichus* sp., *Meyerozyma guilliermondii* and *Pseudeurotium ovale* (Ascomycota: 0.975% in the soil, 2.504% in the wood), and *Malassezia* spp. (Basidiomycota: 0.16% in the soil, 0.001% in the wood).

The aquatic fungi present in the soil and wood included 11 taxa: *Aureobasidium melanogenum*, *Halenospora* spp., *Lemonniera terrestris*, *Minutisphaera parafimbriatispora*, *Mycofalcella calcarata*, *Pleotrichocladium opacum*, *Tricladium splendens*, *Zalerion* sp. and *Zopfiella* spp. (Ascomycota: 0.041% in the soil, 0.527% in the wood), *Cystofilobasidium* spp. and *Phloeomana speirea* (Basidiomycota: 0.012% in the soil, 0.025% in the wood).

The rock-inhabiting fungi included one taxon, *Capnobotryella renispora* (Ascomycota: 0.005% in the soil).

The individual fungi often belonged to more than one trophic group.

Margalef’s index (*D*_Mg_), Shannon’s diversity index (*H*) and Simpson’s diversity index (*D*) indicated greater diversity in the soil than in the wood. Shannon’s evenness index (*E*) showed more evenness in the soil and, conversely, Berger-Parker’s dominance index (*d*) showed more dominance of individual taxa in the wood.

## 4. Discussion

### 4.1. Disease Characteristics

The vascular wilt of hybrid poplar appeared locally in Poland in 2017. The symptoms appeared suddenly in 5–6-year-old trees, and the disease developed very quickly, in less than 2 months. The activity of the pathogens, either already known or previously unrecognized, apparently circumvented any resistance in the host and led to the failure of the plantations. The disease was asymptomatic in its initial stage. Diagnosis at the final stage was not possible because of either: (i) the immaturity of the pathogen, or (ii) the absence of the distinctive morphological elements essential for the identification of causal fungi. Poplar diseases have a serious economic impact on wood production worldwide, and so the development of effective management strategies depends on the clear identification of the pathogens involved. The affected tissues were therefore analyzed by DNA sequencing.

The symptomatology of poplar wilt can be compared with that of some grapevine diseases, notably grapevine trunk diseases (GTD), including the esca and black foot diseases, and Petri disease [14,15]. Grapevine trunk disease symptoms include the sectorial and/or central necrosis of the trunk wood, brown streaking of the wood, cankers, and the discoloration and wilting of the foliage, which can occur suddenly [15,16]. Petri disease is a vascular disease associated with the decline and dieback of young grapevines. Typical black foot disease symptoms include stunted growth, reduced vigour, retarded or absent sprouting, sparse and chlorotic foliage with necrotic margins, wilting, dieback and death. Characteristic sunken necrotic root lesions with a reduction in root biomass and root hairs may also occur.

Grapevine trunk disease is caused by fungi in the Botryosphaeriaceae [17,18], *Phomopsis viticola* [17,19], *Eutypa lata* [20] and *Truncatella* [21]. Petri disease and esca are caused by six species of *Cadophora*, including *C. luteo-olivacea*, 29 species of *Phaeoacremonium* (*particularly P. cinereum*), *Phaeomoniella chlamydospora* (Gams, Crous, Wingf. and Mugnai) Crous and Gams, *Pleurostoma richardsiae* (Nannf.) Réblová and Jaklitsch (=*Phialophora richardsiae* (Nannf.) Conant), and basidiomycetous *Fomitiporia mediterranea* (Fisch.) and *Stereum hirsutum* (Willd.) Pers. [15,22,23,24,25]. Black foot disease is caused by species of *Campylocarpon*, *Cylindrocladiella*, *Dactylonectria*, *Ilyonectria*, *Neonectria* and *Thelonectria* [26]. The fungal species associated with grapevine diseases, mentioned above, have also been reported from a broad range of woody and herbaceous host plants [23,27,28,29,30]. In Italy, *Cadophora*, *Coniochaeta* (in its *Lecythophora* anamorphic stage) and *Phaeoacremonium* have been isolated from the wood of kiwifruit plants suffering from elephantiasis, which had trunk necrosis, hypertrophy and longitudinal bark cracks [31].

### 4.2. Pathogens in Diseased Poplar Trunk

According to EN 350:2016, poplar wood is non-durable, and some studies have shown that it is highly susceptible to wood-rotting fungi [32,33].

The dominant taxonomic group of poplar-associated fungi was Ascomycota. Those fungi are often cosmopolitan species known from the above- and below-ground parts of *Populus* species. Many species found in the wood of diseased trees are, however, known from diseased grapevine: Botryosphaeriaceae, *C. luteo-olivacea*, *Dactylonectria* spp., *Ilyonectria* spp., *Neonectria* spp., *P. cinereum*, *Phaeomoniella* spp., *Phialophora* spp., *Phomopsis* spp., *Thelonectria* spp. and *Truncatella* spp. Other vascular and parenchymal fungi, frequently necrotrophic species, were also found: *Angustimassarina*, *Aureobasidium*, *Boeremia*, *Chaetomium*, *Chaetosphaeria*, *Cyathicula*, *Cudoniella*, *Dendryphion*, *Didymella*, *Fusarium*, *Graphium*, *Helicodendron*, *Helicosporium*, *Hymenoscyphus*, *Hypoxylon*, *Knufia*, *Leptodontidium*, *Leptosphaeria*, *Lophiostoma*, *Massarina*, *Megacapitula*, *Mollisia*, *Neocatenulostroma*, *Neoleptosphaeria*, *Neosetophoma*, *Niesslia*, *Ophiostomatacea* (with its anamorphs), *Phoma*, *Plagiostoma*, *Pleurophoma*, *Podospora*, *Pyrenochaeta*, *Scutellinia*, *Scytalidium*, *Sporothrix*, *Tricharina*, *Xenopolyscytalum*, *Verticillium*, and basidiomycetous *Burgoa*. These fungi were also often in the surrounding soil. Some of them seem likely to have contributed to the disease-causing species complex. The fungi associated with the diseased poplars, and which had been found previously in the wood of poplar or other deciduous trees, included: *Angustimassarina* on the wood of grapevine and poplar [34], *Chaetosphaeria* on the necrotic wood of *Prunus* [35], *Graphium penicillioides* in a wood core of *Populus nigra* in the Czech Republic 200 years ago [36], *Graphostroma platystomum* on the bark of oak [37], *Helicodendron luteoalbum* on poplar roots [38], *Helicosporium* on a wilted chestnut tree [39], and *Hymenoscyphus caudatus* on the rotten leaves of *Populus nigra* [40]. The last species is related to *Hymenoscyphus fraxineus* (T. Kowalski) Baral, Queloz and Hosoya, which causes a very destructive wilt disease of ash, ash dieback—with similar trunk symptoms to those observed in the hybrid poplar [41,42]. *Infundichalara microchona* occurred in conifers [43,44]; *Knufia* in black galls on the stems and branches of *Populus tremuloides* Michx. in Canada [45]; *Leptodontidium* on the roots of healthy *Populus deltoides* [46]; *Lophiostoma corticola* on the above-ground organs of dying oaks in Poland [47]; *Megacapitula* on fallen, decaying petioles of broad-leaves trees [48]. *Mollisia* occurred on decaying plant tissues throughout the Northern Hemisphere; *Neocatenulostroma germanicum* in oak-wood debris [49]; *Neoleptosphaeria rubefaciens* occurred on the wood, bark and fruits of herbaceous or woody plants in terrestrial habitats [50,51,52]. *Neosetophoma clematidis* occurred on the branches of *Clematis vitalba* L. [53] and *Niesslia mucida* on the bark of diverse plants, especially conifers [54]. Ophiostomataceae have been associated with wounds on hardwood trees in Poland [55]. *Phaeoacremonium* species occurred on European olive, quince and willow [27]; *Phialocephala* on rotten deciduous wood [56]; *Phoma* on the decaying wood of oak and pine [57]; *Plagiostoma* in the stems, twigs, and branches of woody and herbaceous plants from a wide range of plants in temperate regions of the Northern Hemisphere [58,59]. *Pleurophoma ossicola* occurred in Scots pine [60], and *Pyrenochaeta* occurred in oak [57]. *Scytalidium lignicola* causes diseases in *Citrus* and *Manihot* [58,61,62]. *Sporothrix* occurred in eucalyptus, pine and rosebush [63], and *Xenopolyscytalum pinea* in pine stumps [64].

Basidiomycetous *Burgoa anomala* was found in pine wood and litter [65].

Some of the fungi are, surprisingly, often common on wood in water, including sea water. This group includes *Didymosphaeria futilis*, *Halenospora varia*, *Halosphaeria quadriemis*, *Paraphoma radicina*, *Trichocladium* and basidiomycetous *Cystobasidium* [66,67,68,69,70,71,72]. *Fusarium* spp. were not abundant in the poplar wood, but occurred frequently in the soil. Various *Fusarium* spp. have been reported in Poland as causing swellings, necrosis, bark fray, reddish-purple discoloration, and ultimately the characteristic cankers in poplar [73]. *Fusarium avenaceum* is perhaps the most important species, first reported in the 1950s on Euramerican poplar clones in France. Since then it has spread in Europe, from central and eastern areas with a continental climate to sub-mediterranean areas, and recently to Portugal, with its oceanic climate. *Neocosmospora solani* (=*Fusarium solani* (Mart.) Sacc. (found mostly on Aigeiros and Tacamahaca poplars and intersectional hybrids) seemed to be confined to North America until it was reported in Poland [74]. Species with sporadic occurrence and of limited importance include *F. lateritium* Nees, observed in France and in the USA on *Populus trichocarpa* Torr. and A. Gray, and *F. sporotrichioides* Sherb., observed in eastern Europe and central Italy on *Populus* × *euramericana*. *Fusarium* spp., constituting a threat to young trees. Colonized trunks are susceptible to breakage, and to attacks by other bark parasites which are also active during a plantation’s early years. The symptoms are not immediately visible, and mostly take the form of the disorganization of the cortical tissues in part of the trunk.

Fungi which are more frequent and perhaps more significant than *Fusarium* spp. in diseased poplar wood include *Cytospora*, *Diaporthe* (with its *Phomopsis anamorph*), *Graphium*, *Ilyonectria*, *Paraphoma*, *Phaeoisaria* and *Phialophora*.

*Cytospora* species are cosmopolitan, facultative parasites, and appear in tree stands subjected to some form or stress, with poor agronomic management or infected by other pathogens. Infection occurs in late autumn or winter, when the host is dormant, usually behaving as a distinctly secondary parasite. The initial symptoms include brown-blackish discolorations, necrosis, depressions in the bark and underlying wood, callus production and withering. Older, sturdier tissues may develop resistance to further invasion. The disease then appears as small brown depressions bounded by distinct calluses. In the advanced stage, the bark tissues may peel away to reveal underlying stained wood [75]. *Cytospora ambiens*, *C. chrysosperma* and *C. nivea* (Hoffm.) Sacc., which are usually present on/in poplar wood worldwide, with their highest incidence in central and southern Italy, eastern Europe, the Near East, northern India, southern Africa (mainly in plantations) and the west-central USA (especially in Colorado), were not detected in the diseased hybrid poplars.

Species of *Diaporthe* and its *Phomopsis* anamorph comprise a phytopathologically important group, with diverse host associations and worldwide distribution. They cause leaf spots, blights, decay, wilt, root rots, dieback and cankers. *Phomopsis* pathogens are hemibiotrophs, i.e., first latent endophytes requiring living plants as a nutrient source, then sometimes becoming necrotrophic in the latent phase of colonization, or saprotrophic, their nutrients provided by tissue they have killed [76,77]. They occur in both temperate and tropical regions, and are especially common in the sapwood of angiosperms [78,79,80,81,82,83,84,85,86,87,88,89,90,91,92]. Endophytic and saprotrophic strains of *Phomopsis* produce similar degrading enzymes, supporting the thesis that endophytes become saprotrophs at the plant’s senescence [87,93]. *Graphium basitruncatum* has been reported from the gallery of the ambrosia beetle in poplar in South America [94]. *Graphium penicillioides* has been detected in the fully functional, wet sapwood of poplars [36] Baobab. Although the teleomorph of *G. penicillioides* is unknown, the genus is believed to have ophiostomatoid affinities [95,96,97].

*Paraphoma* is root-associated on *Populus*, although *P. chrysanthemicola* has so far been reported only from *Juniperus*, *Malus* and herbaceous plants [97,98].The fungus can infect the leaves of certain plant species and provoke disease [99]. On poplar, it caused foliar blight [100]. The fungus can also live benignly in asymptomatic plant tissues, and has been detected or isolated from the roots of healthy plants [101].

*Phaeoisaria loranthacearum* has so far been reported from twigs of *Loranthus europaeus* in Germany [102].

*Phialophora* species, found very abundantly, may include *P.* richardsiae, a serious pathogen implicated in the Petri disease of grapevine. The significance of other *Phialophora* spp. potentially occurring in the diseased poplar wood should also be emphasized. They are mostly saprotrophic and common in soil and wood, in which they cause soft rot. Growth at the hyphal tip and the secretion of lignolytic enzymes (pectinase, amylase, xylanase, cellulase and mannanase) causes widened cavities in sapwood and the degradation of the wood [103,104]. They can also cause cavities in the wood and plants via an erosion-type attack [105]. The degradation of *Populus tremuloides* wood has been known to affect sales of commercial aspen timber. The blue staining of wood by *Phialophora* has also been reported [106]. The fungus is psychrotolerant (able to grow at a low temperature).

Many of the taxa recorded, especially in the soil, may not be poplar-specific. They would originate from nearby vegetation, litter and decaying organic matter. Ascomycetous *Boeremia* spp., *Desmazierella acicola*, *Dissoconium eucalypti*, *Entyloma gaillardianum*, *Lambertella tubulosa*, *Leptosphaerulina australis*, *Microdochium* sp., *Monographella nivalis*, *Neosetophoma clematidis*, *Periconia* sp., *Phacidium* spp., *Phaeosphaeria* sp., *Phaeosphaeriopsis* sp., *Phialocephala* sp., *Pyrenochaetopsis* spp., *Schizothecium glutinans*, *Xenochalara* sp., *Xenopolyscytalum* spp., *Xenoramularia arxii*, and basidiomycetous *Aecidium* sp., *Entyloma* spp. and *Itersonilia perplexans* possibly spread from weeds, grass roots, leaf litter and woody debris [107,108,109,110,111,112,113,114,115,116,117,118,119,120,121]. *Neocatenulostroma germanicum*, recently found in Europe, seems to spread from pine needles or oak wood debris [49,122].

The cosmopolitan *Cenococcum geophilum*, one of the most frequently encountered ectomycorrhizal fungi in nature, is well recognized for its extremely wide host and habitat range [123].

Fungi of the genera *Alternaria*, *Epicoccum*, *Fusarium*, *Cladosporium*, *Penicillium* and *Trichoderma* are highly robust and ubiquitous, with an almost global distribution, occurring in the Americas, Asia, and Europe [103]. Their spores have been found in a variety of habitats, predominantly in soil of various types and in sand, often in extreme conditions. *Epicoccum* can grow on leaves submerged in water, even at 0 °C; hyphal growth can resume within an hour of exposure to water [104,124].

Some fungi were recorded for the first time on wood, or have been found rarely on wood. Ascomycetous *Neocatenulostroma germanicum* is known from pine needles, and is known to cause needle blight on *Pinus mugo* Turra, *P. nigra* Arn. ssp. *pallasiania* and *P. sylvestris* L. in Lithuania, Poland and Ukraine [44,122], but has also occurred in the soil in Poland [125]. *Sydowia polyspora* is so far known from the foliage of *Abies* spp., *Pinus* spp. and *Pseudotsuga menziesii* (Mirb.), and litter [126]. Research suggests that some of these hosts can be primary inoculum sources when located near poplar plantations [127].

Some more- or less-frequent colonizers are untypical and dubious. *Acaulium retardatum* has so far been recorded from rice-field soil [128], *Acrodontium crateriforme* from trap-liquid of pitcher plant *Nepenthes khasiana* Hook f. A.L.P.P. de Candolle, Prodr. in India [129], *Alatospora* has been recorded from aquatic habitats [130], *Amesia nigricolor* has been recorded from an indoor habitat in India [131], *Cercospora beticola* from sugar beet leaves, *Desmazierella acicola* from pine needle litter [132,133], *Dissoconium eucalypti* from *Eucalyptus* leaf [134], *Halokirschsteiniothelia maritima* from decaying wood in Thailand [135], *Nigrospora oryzae* from tropical plants [136], *Pleurophoma ossicola* from bone [102], *Pseudocercospora angolensis* from leaf spot on *Citrus* in Africa [137], *Sakaguchia lamellibrachiae* (Nagah., Hamam., Nakase and Horikoshi) Wang, Bai, Groenew. and Boekhout from a deep-sea tubeworm in Japan [138], and the basidiomycetous yeast *Erythrobasidium hasegawianum* has been recorded from old beer yeast culture in USA [139].

Some can occur at the extreme of their host ranges. *Graphium basitruncatum* has been isolated from wood and soil, even in the Solomon Islands and Japan, and from a leukemic patient [140,141]. *Scytalidium lignicola* and *Sporothrix* are recognized as saprotrophic opportunists of which the lifestyle can change from plant to human or animal pathogenicity.

Oomycota with eight species of *Globisporangium*, two species of *Phytophthora* and eight species of *Pythium* were mostly in the soil, and were not very common. Their contribution to the development of the disease cannot be excluded. All of them are plant pathogens, which cause root rot and damping off in a multitude of species. *Phytophthora plurivora* Jung and Burgess, followed by *P. pini* Leonian, *P. polonica* Belbahri, E. Moralejo, Calmin and Oszako, *P. lacustris* Brasier, Cacciola, Nechw., Jung and Bakonyi, *P. cactorum* (Lebert and Cohn) Schröt, and *P. gonapodyides* (Petersen) Buisman. were common in three declining and three healthy poplar plantations in Serbia [142].

### 4.3. Yeasts in Diseased Poplar Trunks

Yeasts are now identified and classified almost exclusively by DNA sequence analysis, which has resulted in the discovery of many new species and taxonomic revisions.

Filamentous fungi have a key role in the decomposition of plant material because of their ability to produce a wide range of extracellular enzymes that efficiently attack the recalcitrant lignocellulose matrix. However, the presence of yeasts during the different stages of wood breakdown highlights the ecological role of these microorganisms. Yeasts have been found to produce enzymes acting on cellulose, hemicelluloses and pectin [143]. They can therefore degrade plant material. They can also be transient fungi, using products released during decomposition by other organisms. Many yeast species found in live or decaying plant parts are associated with insects that also use these habitats as feeding or breeding sites.

The general opinion is that the most abundant yeast taxa associated with decayed wood are basidiomycetous (Agaricomycotina) and xylose-assimilating species. The present data do not support this thesis. Some ascomycetous yeasts were particularly abundant in the wood, where basidiomycetous yeasts were much less frequent.

Ascomycetous *Aureobasidium pullulans* and *Candida* spp., and basidiomycetous species of *Apiotrichum*, *Cystofilobasidium*, *Naganishia*, *Saitozyma*, *Solicoccozyma*, *Tausonia*, *Tremella*, *Trichosporon* and *Vishniacozyma* are frequently found in decaying plant material [143]. However, variations in their abundance and diversity reflect the environment, and also correlate with the natural abundance and distribution of basidiomycetous fungi in the study areas [144]; *Apiotrichum*, for example, was reported as being abundant in wood decayed by *Armillaria*. The abundance of ascomycetous yeasts in the wood resulted from the high frequency of *Nakazawaea* spp., especially *N. populi*, which was previously found in exudates of *Populus* species [145].

### 4.4. Mycorrhiza-Forming Fungi

Mycorrhiza-forming fungi were rare, especially in the soil. Basidiomycetous species occurred, surprisingly, more often in the wood, probably as: (i) facultative biotrophic encounters that either formed mycorrhizal structures or colonized the tissues as endophytes (i.e., grew within living plant tissues, without apparent infection, but not forming true mycorrhizae or causing any disease symptoms), or (ii) saprotrophs. Transition from saprotrophy to mycorrhizal status is common in fungal development [146], and other unexpected trophic conversions within the mycobiota may be possible.

### 4.5. The Endophytic State/Habit/Lifestyle of Fungi

As with grapevine diseases, it is assumed that the causal fungi are endophytic, living for a time asymptomatically in the plant. Then, at some point, in association with plant stress, they modify their behaviour and become pathogenic, which leads to the expression of disease symptoms [147]. As endophytes, they would often have key positive roles in plant function and fitness [148,149]. As parasites, they are cryptic, often opportunistic pathogens, which in special conditions induce disease [150]. Their virulence may be dictated by multi-partner interactions and environmental conditions. The most favoured conditions include: (i) the presence of very vigorous plants with succulent tissues; (ii) prolonged periods of damp and wet weather; (iii) free-standing water on the leaves; (iv) injuries such as pruning and leaf wounds; (v) the presence of senescent tissues, especially older, lower leaves; (vi) frost damage; and (vii) excessive crowding. Tissues are invaded by enzyme action, and roots and stems are gradually enveloped until the vessels are eventually reached, and wilting and desiccation occur. Different lifestyles and functions may occur depending on the situation. *Phoma* may at first be a plant-growth­–promoting fungus [151].The lifestyles of *Phaeoisaria* and *Pyrenochaetopsis* depend on secreted peptidases [121,152]. *Plectosphaerella* (mostly *P. populi*) damages poplar stems [102,152], but simultaneously induces the formation of antifungal phenolic metabolites that protect poplar against foliar pathogens [153]. Some, such as *Pyrenochaeta*, are weak pathogens [154], but their adaptability to different climates allows them to infect many hosts and to survive in a broad range of pH, temperature and aeration conditions and soil types. Fungi such as *Ilyonectria* may survive in the roots of apparently healthy (asymptomatic) poplars, where they may suppress other fungal root pathogens and help maintain tree health [27,30]. These examples show that caution is necessary in classifying fungi according to function. There is no indication that other species, uncommon on *Populus* or so far not detected, might be pathogenic.

### 4.6. Interactions among Fungi

*Trichoderma* spp. occurred at a high natural frequency in the plantation soil. They are well known for their antagonistic activity, hyperparasitism and ability to induce defensive systems in plants to other microorganisms (specifically soil microorganisms). They are used in the biological control of several pathogens. *Trichoderma harzianum* Rifai and *T. atroviride* Karst. have shown promise in controlling Botryosphaeria dieback and esca disease in vineyards and other common trunk diseases [155]. *Trichoderma* significantly improved grapevine root growth and decreased the incidence of fungi involved in diseases when tested in vitro or in nurseries [24,156]. Grapevine defence systems have also been induced by Oomycota. The necrosis of root systems of vine cuttings was reduced by 50% after colonization by *Pythium oligandrum* [157,158,159]. Other biological control agents (*Aureobasidium pullulans*, *Cladosporium herbarum*, *Fusarium lateritium* and *Rhodotorula rubra*) have been reported to be effective against grapevine trunk disease pathogens, alone or in combination with fungicides, although some were tested only in vitro or in nurseries [160]. Arbuscular mycorrhizal fungi have been shown to increase the tolerance of grapevine rootstocks to *Ilyonectria* spp. [161]; *Glomus intraradices* was the most effective [162]. *Aureobasidium pullulans*, *P. oligandrum*, *Trichoderma* spp. and two species of Glomeromycota, present in the poplar plantation soil, may naturally decrease the incidence of pathogens involved in disease. *Mortierella elongata*, also detected, has been found to manipulate poplar defenses while promoting plant growth [30].This response was particularly beneficial because it was independent of cultivars.

### 4.7. Soil and Planting Material as the Source of the Inoculum

The soil origin was shown to be a significant factor affecting the composition of the fungal communities and networks in *Populus* [149,163].

The soil was here shown to be a natural source of many vascular and parenchymal pathogens found in the affected hybrid poplars, i.e., species of ascomycetous *Alternaria*, *Cadophora*, *Cladosporium*, *Fusarium*, *Ilyonectria*, *Nectria*, *Neonectria*, *Neopyrenochaeta* Ophiostomataceae, *Phoma*, *Pyrenochaeta*, *Sporothrix*, *Thelonectria* and *Verticillium*, and of basidiomycetous *Armillaria* and *Entyloma*. Their presence in the soil has been associated with their occurrence on plant debris and plant roots [164]. Soil was also the main source of pathogenic Oomycota (*Aphanomyces*, *Elongisporangium*, *Globisporangium*, *Phytophthora* and *Pythium*), which can, generally, cause extensive and devastating root rot. The destruction of roots can lead to minor or severe wilting caused by impeded root functioning or further biotrophic infections that can become necrotrophic in response to infection pressure or environmental stress. Oomycota tend to be very generalistic and non-specific, with a wide range of susceptible host roots, including poplar [142]. The wilt results from root degradation by Oomycota and a lack of oxygen, followed by disrupted water transport. A moist habitat and low pH in forest soils favour the growth, propagation, and dispersal of Oomycota spores. At optimal temperatures (28–30 °C), some species of *Globisporangium* grow very fast, i.e., 2.7 cm in 24-h.

Fungi such as *Collophorina*, *Hyalodendriella* and *Hyaloscypha bicolor*, which occurred sporadically in the soil, whilst being biotrophic parasites, may contribute to the final wilt [165,166].

The planting material may, however, already have been infected, either systemically from infected mother poplars or by contamination during the propagation process.

### 4.8. Colonization

As in grapevine disease, poplar wilt may be a complex disease in which symptoms result from the concomitant action of several factors.

The initial stage of the disease seems to be accomplished by highly specialized vascular fungi in the plant’s phloem. Their presence in the soil suggests that the infection can be soil-borne. Hyphae from established mycelia, and germ tubes developing from spores, perceive signals from root exudates. The hyphae secrete cell-wall–degrading enzymes and enter roots through wounds, at branching points, or directly through root tips. The mycelium spreads between root cortex cells to reach phloem and xylem vessels, from which the fungus travels as conidia in the sap stream, mostly upwards. The phloem and xylem become obstructed by mycelium and spores, and by plant-produced gels, gums and tyloses. Water transport to the leaves fails, and the plant wilts and dies. The fungus then invades all of the plant tissues and obtains nutrition by decomposing them. The response to the degradation of hemicellulose or lignin by the pathogen is usually the accumulation of tylose, polysaccharides and phenolic compounds (gummosis), tannins and phytoalexins. It is likely that at least a part of the external and internal symptoms are caused by phytotoxic fungal metabolites produced in decayed wood, or by the oxidation of some host-response substances. Some chemicals produced in grapevine in response to fungal infection are toxic, notably α-glucans and two naphthalenone pentaketides, scytalone and isosclerone [22]. A similar situation may be expected in poplar.

The final stage of the disease is apparently accomplished by parenchymal fungi. The spores released from reproductive structures produced in dead wood in the presence of water are dispersed by wind, potentially infecting fresh new wounds. Among the parenchymal fungi, bracket fungi (Polyporales, Basidiomycota) were, surprisingly, found only sporadically; they usually dominate communities of wood-rotting organisms. In grapevine, the phytoalexin resveratrol showed a direct antifungal effect, inhibiting the in vitro growth of two bracket species, *Fomitiporia mediterranea* and *Stereum hirsutum*. It is possible that the accumulation of certain compounds produced by poplar suppresses the colonization of wood by bracket fungi.

### 4.9. Effects of Climate

Up to 133 fungal species of 34 genera have so far been associated with grapevine trunk diseases worldwide [127]. The incidence of particular taxa differs between regions. All known grapevine trunk pathogens have been encountered in all grape-cultivation regions, mainly between latitudes of 30° to 50°, where annual mean temperatures are generally 10–20 °C [127,167]. There are conflicting reports on the effects of temperature and water stress on the incidence of grapevine trunk disease [127]. Therefore, it is not possible to assume a straightforward relationship between poplar disease and climatic conditions, particularly concerning water stress. Water stress is likely, however, to increase susceptibility. In recent years, precipitation in central Europe has often been characterized by extreme events (fog, hailstorms, thunderhails, heat waves, heavy rains, floods, winds), followed by drought. Increased humidity favours disease development. Infection by ascospores or conidia released from perithecia or pycnidia embedded in the bark or wood will be promoted by high humidity, often associated with higher temperatures; such conditions encourage the release and spread of spores, and favour spore germination [168,169,170,171]. The inoculum potential is consequently increased.

An extremely hot and dry summer (particularly August and September) occurred across Poland in 2015. The climate projections for Poland and central Europe predict further warming and the continuation of the changes already observed, including decreased precipitation and drought, especially in summer [172]. Such conditions may be expected to affect the health of poplar and other trees.

### 4.10. Control and Mitigation

Fungicides such as sodium arsenite or 8-hydroxyquinoline, used against esca and with the potential to control the wilt of poplar, are banned in Europe. No other highly effective treatments are available. Other chemical products and biological stimulators used in vineyards are not curative, and so only preventive methods are available in poplar plantations. Infections in grapevine from propagating materials can increase from 40% before cuttings are taken up to 70% after nursery processing [172]. Detection prior to planting is therefore critical to assure the longevity of newly established plantations [173]. A healthy poplar at planting is fundamental to the establishment and sustainability of a plantation. Good hygiene and wound protection are of the utmost importance. The disinfection of propagating materials with fungicides or hot water treatment (50 °C for 30 min), applied correctly to avoid plant stress and death, is advisable. Where soil constitutes the main source of the inoculum, disease management practices based on soil disinfestation and amendments, plant-based resistance to infection, and prophylactic cultural practices should be applied. Infected plant parts and infected dead wood on the soil should be removed, pruning wounds should be chemically protected, and the elimination of plant-stress factors should be taken into account.

## 5. Conclusions

1. *Populus* hybrids may be subjected to various, thus far unidentified pathogenic agents.
2. New diseases may be asymptomatic, at least in the initial phase.
3. The indigenous microbiota can be involved in the development of the disease, but can also have an important role in limiting or preventing the development of pathogens.
4. The development of new diseases is related to climate change. It can lead to the near-total disappearance of some diseases, the sudden emergence of a new pathogens, or to the fungi already present becoming pathogenic.
5. Poplar wilt symptoms may be a consequence of various factors, the most important being climate and its effects on fungal development and the host–pathogen relationship.
6. Fungal diseases can spread from the soil or from introduced plant material, with the latter potentially introducing them into new areas.

## Figures and Tables

**Table 1:** Microbiota present in the soil and wood of the diseased poplar.

No.	Taxon	Order	Soil	Wood	Trophic Group
**Chromista**					
**Oomycota**					
1.	*Aphanomyces* spp.	Saprolegniales	0.042		Pathogens
2.	*Elongisporangium anandrum* (Drechsler) Uzuhasi, Tojo & Kakish	Peronosporales	0.004		Pathogen
3.	*Globisporangium apiculatum* (B. Paul) Uzuhashi, Tojo & Kakish. + G. *heterothallicum* W.A. Campb. & F.F. Hendrix + *G. intermedium* (de Bary) Uzuhashi, Tojo & Kakish. + *G. macrosporum* (Vaartaja & Plaäts-Nit.) Uzuhashi, Tojo & Kakish. + *G. mamillatum* (Meurs) Uzuhashi, Tojo & Kakish. + *G. pleroticum* (Takesi Itô) Uzuhashi, Tojo & Kakish. + *G. sylvaticum* (W.A. Campb. & F.F. Hendrix) Uzuhashi, Tojo & Kakish. + *G. ultimum* (Trow) Uzuhashi, Tojo & Kakish	Peronosporales	1.010	0.001	Pathogens
4.	*Hyaloperonospora cochleariae* (Gäum.) Göker, Riethm., Voglmayr, Weiss & Oberw	Peronosporales	0.017		Pathogen
5.	*Isoachlya intermedia* (Coker & J.V. Harv.) Coker	Saprolegniales	0.007		Saprotroph
6.	*Myzocytiopsis* sp.	Peronosporales	0.005		Nematopathogenic
7.	*Phytophthora brassicae* De Cock & Man in ‘t Veld + *P. citricola* Sawada + *P. clandestina* P.A. Taylor, Pascoe & F.C. Greenh	Peronosporales	0.040		Pathogens
8.	*Pythium conidiophorum* Jokl. + *P. oligandrum* Drechsler + *P. pachycaule* Ali-Shtayeh + *P. selbyi* M.L. Ellis, Broders & Dorrance + *P. vanterpoolii* V. Kouyeas & H. Kouyeas + *P. volutum* Vanterp. & Truscott + *Pythium* spp.	Peronosporales	0.053	0.001	Pathogens
9.	*Thraustotheca clavata* (de Bary) Humphrey	Saprolegniales	0.021		Saprotroph
**Frequency Oomycota**			**1.199**	**0.002**	
**Number of taxa Oomycota**			26	2	
**Fungi**					
**Blastocladiomycota**					
**Frequency Blastocladiomycota**			**0.005**		
**Number of taxa Blastocladiomycota**			1		
**Chytridiomycota**					
1.	Chytridiomycota		0.004		
2.	*Rhizophydium* sp.	Rhizophydiales	0.004		Pathogen
**Frequency Chytridiomycota**			**0.008**		
**Number of taxa Chytridiomycota**			2		
**Glomeromycota**					
1.	*Entrophospora* sp.	Diversisporales		0.001	
**Frequency Glomeromycota**				**0.001**	Mycorrhizal
**Number of taxa Glomeromycota**				2	
**Zygomycota**					
1.	*Mortierella alpina* Peyronel + *M. amoeboidea* W. Gams + *M. antarctica* Linnem. + *M. elongata* Linnem. + *M. epicladia* W. Gams & Emden + *M. exigua* Linnem. + *M. fatshederae* Linnem. + *M. gamsii* Milko + *M. horticola* Linnem. + *M. humilis* Linnem. + *M. hyalina* (Harz) W. Gams + *Mortierella* spp.	Mortierellales	3.483	0.006	Saprotrophs
2.	Mortierellales	Mortierellales	0.006		
3.	*Mucor racemosus* Bull.	Mucorales	0.012		Saprotrophs
4.	*Ramicandelaber* sp.	Kickxellales	0.004		
5.	*Rhizopus arrhizus* A. Fisch. + *R. oryzae* Went & Prins. Geerl.	Mucorales	0.019		
6.	*Syncephalis* sp.	Zoopagales	0.107		Mycoparasite
**Frequency Zygomycota**			**3.631**	**0.006**	
**Number of taxa Zygomycota**			18	3	
**Ascomycota**					
1.	*Acaulium retardatum* (Udagawa & T. Muroi) Lei Su	Microascales	0.004		Saprotroph
2.	*Acericola italica* Wanas., Camporesi, E.B.G. Jones & K.D. Hyde	Pleosporales		0.001	
3.	*Acremonium persicinum* (Nicot) W. Gams + *A. rutilum* W. Gams	Hypocreales	0.001	0.002	Saprotrophs
4.	*Acrodontium crateriforme* (J.F.H. Beyma) de Hoog	Incertae sedis	0.013		
5.	*Alatospora acuminata* Ingold + *Alatospora* sp.	Helotiales	0.113	0.026	
6.	***Alternaria alternata* (Fr.) Keissl. + *A. botrytis (Preuss) Woudenb.* & Crous + *A. infectoria* E.G. Simmons + *A. tenuissima* (Kunze) Wiltshire + *Alternaria* sp.**	Pleosporales	0.065	0.039	Pathogens
7.	*Amesia nigricolor* (L.M. Ames) X. Wei Wang & Samson	Sordariales	0.001		Saprotroph
8.	*Angustimassarina acerina* Jayasiri, Thambug., R.K. Schumach. & K.D. Hyde + *A. populi* Thambug. & K.D. Hyde	Pleosporales	0.354		Mycoparasite
9.	Arthoniomycetes		0.001	0.001	
10.	*Ascobolus* sp.	Pezizales	0.005		Saprotroph, coprophilous
11.	*Ascochyta skagwayensis* (R. Sprague) Punith.	Pleosporales		0.001	Saprotroph, pathogen
12.	Ascomycete			0.027	
13.	Ascomycota		1.123	0.215	
14.	*Aspergillus conicus* Blochwitz + *A. niger* Tiegh. + *A. penicillioides* Speg. + *A. versicolor* (Vuill.) Tirab.	Eurotiales	0.008	0.003	Saprotrophs
15.	*Atrocalyx lignicola* (Ying Zhang, J. Fourn. & K.D. Hyde) A. Hashim. & Kaz. Tanaka	Pleosporales		0.009	Saprotroph
16.	*Aureobasidium melanogenum* (Herm.-Nijh.) Zalar, Gostinčar & Gunde-Cim. + *A. pullulans* (de Bary & Löwenthal) G. Arnaud + *Aureobasidium* sp.	Dothideales	0.003	0.013	Saprotrophs, often aquatic
17.	*Bacidina* sp.	Lecanorales		0.018	Lichenicolous
18.	*Beauveria bassiana* (Bals.-Criv.) Vuill. + *Beauveria* sp.	Hypocreales	0.049	0.002	Entomopathogenic
19.	*Blastobotrys malaysiensis* Kurtzman + *Blastobotrys* sp.	Saccharomycetales	0.009	0.013	Saprotrophs
20.	***Boeremia exigua* (Desm.) Aveskamp, Gruyter & Verkley + *B. noackiana* (Allesch.) Gruyter & Verkley**	Pleosporales	0.006	0.017	Pathogens
21.	***Cadophora luteo-olivacea* (J.F.H. Beyma) T.C. Harr. & McNew + *C. spadicis* Travadon, D.P. Lawr., Roon.-Lath., Gubler, W.F. Wilcox, Rolsh. & K. Baumgartner + *Cadophora* sp.**	Helotiales	0.114	1.435	Pathogens
22.	*Candida sake* (Saito & M. Ota) Uden & H.R. Buckley ex S.A. Mey. & Ahearn + *C. subhashii* M. Groenew., Sigler & S.E. Richardson + *C. vartiovaarae* (Capr.) Uden & H.R. Buckley + *Candida* sp.	Saccharomycetales	0.093	0.012	Saprotrophs
23.	*Capnobotryella renispora* Sugiy	Capnodiales	0.005		Saprotroph
24.	Capnodiales	Capnodiales	0.017		
25.	*Cenococcum geophilum* Fr.	Mytilinidiales	0.039		Ectomycorrhizal
26.	Cephalothecaceae	Sordariales	0.003		Saprotrophs, mycoparasites
27.	Ceratostomataceae	Melanosporales	0.004		Saprotrophs, mycoparasite
28.	*Cercophora* sp.	Sordariales	0.014		Coprophilous
29.	***Cercosporabeticola* Sacc.**	Capnodiales		0.012	Pathogen
30.	Chaetomiaceae	Sordariales	0.085		Saprotrophs
31.	*Chaetomium globosum* Kunze + *Ch. piluliferum* J. Daniels + *Chaetomium* sp.	Sordariales	0.062	0.002	Saprotrophs, endophytes
32.	*Chaetosphaeria vermicularioides* (Sacc. & Roum.) W. Gams & Hol.-Jech.	Chaetosphaeriales	0.005		Saprotroph
33.	Chaetothyriales	Chaetothyriales	0.104		Parasites of humans and cold-blooded animals
34.	*Chalara microspora* (Corda) S. Hughes + *Chalara* sp.	Helotiales	0.007	0.001	Saprotroph
35.	*Chloridium paucisporum* C.J.K. Wang & H.E. Wilcox	Helotiales		0.001	Ectendomycorrhizal
36.	*Chrysosporium pseudomerdarium* Oorschot	Onygenales	0.004		Endophyte
37.	*Cistella albidolutea* (Feltgen) Baral	Helotiales	0.003		Saprotroph
38.	*Cladophialophora minutissima M*.L. Davey & Currah + *Cladophialophora* sp.	Chaetothyriales	0.002		Saprotrophs, human pathogens
39.	*Cladorrhinum flexuosum* Madrid, Cano, Gené & Guarro	Sordariales	0.008		Saprotroph
40.	***Cladosporium allicinum* (Fr.) Bensch, U. Braun & Crous + *C. cladosporioides* (Fresen.) G.A. de Vries + *C. colocasiae* Sawada**	Capnodiales	0.096	0.015	Saprotrophs, facultative plant pathogens, mycoparasites
41.	*Clonostachys divergens* Schroers + *C. parva* (Schroers) Rossman, L. Lombard & Crous + *C. rosea* (Link) Schroers, Samuels + *Clonostachys* sp.	Hypocreales	0.187	0.033	Endophytes, mycoparasites
42.	*Coleophoma cylindrospora* (Desm.) Höhn	Helotiales		0.010	Saprotroph
43.	*Collophorina* sp.	Leotiales	0.001		Saprotroph
44.	***Coniochaeta*** **sp.**	Coniochaetales	0.015	0.002	Pathogens, saprotrophs, endophytes, coprophilous, mycoparasite, human pathogens
45.	*Cordyceps bassiana* Z.Z. Li, C.R. Li, B. Huang & M.Z. Fan + *C. brongniartii* Shimazu	Hypocreales	0.047		Enthomopathogenic, mycoparasite
46.	***Cosmospora berkeleyana* (P. Karst.) Gräfenhan, Seifert & Schroers**	Hypocreales	0.027		Saprotroph, pathogen, mycoparasite
47.	*Crocicreas* sp.	Helotiales	0.005		Saprotrophs
48.	Cucurbitariaceae	Pleosporales		0.076	Saprotrophs, pathogens
49.	*Cudoniella indica* J. Webster, Eicker & Spooner	Helotiales		0.002	Saprotroph
50.	*Cyathicula cyathoidea* (Bull.) Thüm	Helotiales	0.006		Saprotrophs
51.	*Cyphellophora sessilis* (de Hoog) Réblová & Unter	Chaetothyriales		0.001	Pathogen
52.	***Cytospora davidiana* Y.L. Wang & X.Y. Zhang + *C. leucostoma* (Pers.) Sacc. + *C. paratranslucens* Norphanph., Bulgakov, T.C. Wen & K.D. Hyde + *Cytospora* sp**.	Diaporthales	0.012	13.720	Pathogens
53.	*Dactylaria dimorphospora* Veenb.-Rijks	Helotiales	0.016		Saprotroph
54.	***Dactylonectria torresensis* (A. Cabral, Rego & Crous) L. Lombard & Crous**	Hypocreales		0.008	Pathogen
55.	***Debaryomyces hansenii* (Zopf) Lodder & Kreger-van Rij**	Saccharomycetales	0.023		Pathogen
56.	*Dendryphion europaeum* Crous & R.K. Schumach. + *D. nanum* (Nees) S. Hughes	Pleosporales	0.268	0.006	Saprotroph
57.	Dermateaceae	Helotiales	0.002		
58.	*Desmazierella acicola* Lib.	Pezizales		0.001	Saprotroph
59.	***Diaporthe cynaroidis* Marinc., M.J. Wingf.** ** & Crous + *D. foeniculina* (Sacc.) Udayanga & Castl. + *D. helicis* Niessl + *D. novem* J.M. Santos, Vrandečić & A.J.L. Phillips + *D. rudis* (Fr.) Nitschke + *Diaporthe* sp.**	Diaporthales	0.017	3.327	Pathogens, endophytes
60.	***Didymella macrostoma* (Mont.) Qian Chen & L. C + *D. pedeiae* (Aveskamp, Gruyter & Verkley) Qian Chen & L. Cai + *D. pinodes* (Berk. & A. Bloxam) Petr. + *D. pomorum* (Thüm.) Qian Chen & L. Cai**	Pleosporales	0.039	0.036	Pathogens
61.	*Didymosphaeria futilis* (Berk. & Broome) Rehm	Pleosporales	0.005		Saprotroph
62.	***Dissoconium eucalypti* Crous & Carnegie**	Capnodiales	0.001		Commensalist, mycoparasite
63.	Dothideomycetes		0.018	0.014	
64.	*Emericellopsis glabra* (J.F.H. Beyma) Backus & Orpurt + *E. minima* Stolk	Hypocreales	0.179		Endophytes
65.	*Endophoma elongata* Tsuneda & M.L. Dave	Incertae sedis	0.005		
66.	***Epicoccum nigrum*** **Link**	Pleosporales	0.002	0.001	Endophyte, saprotroph, pathogen
67.	Eurotiales	Eurotiales	0.001		
68.	Eurotiomycetes		0.002	0.020	
69.	*Exophiala capensis* Crous + *E. equina* (Pollacci) de Hoog, V.A. Vicente, Najafz., Harrak, Badali & Seyedm. + *E. opportunistica* de Hoog, V.A. Vicente, Najafz., Harrak, Badali & Seyedm. + *Exophiala* sp.	Chaetothyriales	0.129	0.031	Saprotrophs, human pathogens
70.	***Fusarium avenaceum* (Fr.) Sacc. + *F. equiseti* (Corda) Sacc. + *F. fujikuroi* Nirenberg + *F. oxysporum* Schltdl. + *F. petersiae* L. Lombard + *F. redolens* Wollenw. + *F. solani* (Mart.) Sacc. + *F. torulosum* (Berk. & M.A. Curtis) Gruyter & J.H.M. Schneid. + *Fusarium* sp.** ** + *Neocosmospora solani* (Mart.) L. Lombard & Crous**	Hypocreales	0.890	0.104	Pathogens
71.	***Fusicolla aquaeductuum* (Radlk. & Rabenh.) Gräfenhan, Seifert & Schroers + * F. merismoides* (Corda) Gräfenhan, Seifert & Schroers**	Hypocreales	0.096		Pathogens
72.	*Gibellulopsis nigrescens* (Pethybr.) Zare, W. Gams & Summerb	Glomerellales	0.009		Saprotroph
73.	*Gliomastix murorum* var. *felina* (Marchal) S. Hughes	Hypocreales	0.023		Saprotroph
74.	***Graphium basitruncatum* (Matsush.) Seifert & G.Okada + *G. penicillioides* Corda**	Microascales	0.007	2.451	Saprotrophs, plant and human pathogens
75.	*Gaphostroma platystomum* (Schwein.) Piroz.	Xylariales	0.004		Saprotroph
76.	*Halenospora varia* (Anastasiou) E.B.G. Jones + *Halenospora* sp.	Helotiales		0.443	Saprotrophs, aquatic
77.	*Halokirschsteiniothelia maritima* (Linder) Boonmee & K.D. Hyde	Mytilinidiales	0.023		Saprotroph
78.	*Halosphaeria quadri-remis* (Höhnk) Kohlm	Microascales	0.007		Saprotroph
79.	Halosphaeriaceae	Microascales	0.008		
80.	*Harzia acremonioides* (Harz) Costantin + *H. sphaerospora* (Matsush.) D.W. Li & N.P. Schultes	Melanosporales	0.028		Saprotrophs
81.	*Helicodendron luteoalbum* Glen Bott + *H. westerdijkiae* Beverw	Helotiales		0.009	Saprotrophs
82.	*Helicosporium* sp.	Tubeufiales		0.006	Saprotrophs
83.	Helotiaceae	Helotiales	0.005		
84.	Helotiales	Helotiales	3.087	4.565	
85.	***Hemibeltrania* sp.**	Amphisphaeriales	0.007		Pathogen
86.	***Herpotrichia pinetorum* (Fuckel) G. Winter + *Herpotrichia* sp.**	Pleosporales	0.183	0.002	Pathogens
87.	Herpotrichiellaceae	Chaetothyriales	0.004		
88.	***Hyalodendriella betulae*** **Crous**	Helotiales	0.012	0.001	Saprotroph, pathogen
89.	*Hyalopeziza* sp.	Helotiales	0.014		Saprotroph
90.	*Hyaloscypha bicolor* (Hambl. & Sigler) Vohník, Fehrer & Réblová	Helotiales	0.012		Endophyte, saprotroph
91.	Hyaloscyphaceae	Helotiales	0.003	0.040	
92.	***Hymenoscyphus caudatus* (P. Karst.) Dennis + *H. imberbis* (Bull.) Dennis**	Helotiales	0.007	0.017	Pathogens, saprotrophs
93.	Hypocreales	Hypocreales	2.979		
94.	***Hypoxylon fragiforme*** (Pers.) J. Kickx f.	Xylariales	0.469	0.002	Saprotroph, pathogen
95.	***Ilyonectria crassa* (Wollenw.) A. Cabral & Crous + *I. cyclaminicola* A. Cabral & Crous + *I. destructans* (Zinssm.) Rossman, L. Lombard & Crous + *I. europaea* A. Cabral, Rego & Crous + *I. mors-panacis* (A.A. Hildebr.) A. Cabral & Crous + *I. robusta* (A.A. Hildebr.) A. Cabral & Crous + *Ilyonectria* sp. + *Cylindrocarpon* sp.**	Hypocreales	2.031	6.710	Saprotrophs, pathogens
96.	*Infundichalara microchona* (W. Gams) Réblová & W. Gams + *I. minuta* Koukol	Helotiales	0.014	0.001	Saprotrophs, patogens, mycoparasitic
97.	*Jattaea taediosa* (Sacc.) Réblová & Jaklitsch	Calosphaeriales		0.005	Endophyte
98.	***Juxtiphoma eupyrena* Sacc.**	Pleosporales		0.001	Pathogen
99.	*Knufia cryptophialidica* L.J. Hutchison & Unter. + *K. peltigerae* (Fuckel) Réblová & Unter	Incertae sedis	0.006	0.015	Pathogens, lichenicolous
100.	*Lambertella tubulosa* Abdullah & J. Webster	Helotiales	1.445		Saprotroph
101.	Lasiosphaeriaceae	Sordariales	0.095	0.005	
102.	*Lecania cyrtella* (Ach.) Th. Fr. + *L. naegelii* (Hepp) Diederich & van den Boom	Lecanorales	0.001	0.034	Lichenicolous
103.	Lecanorales	Lecanorales	0.001		
104.	*Lemonniera terrestris* Tubaki	Helotiales	0.014		Saprotroph, aquatic
105.	*Leohumicola minima* (de Hoog & Grinb.) Seifert & Hambl	Helotiales		0.002	Saprotroph
106.	Leotiomycetes		0.003	0.876	
107.	*Lepraria caesiella* R.C. Harris	Lecanorales	0.002		Lichenicolous
108.	*Leptodontidium* sp.	Helotiales	0.011	0.254	Endophyte, mycorrhizal
109.	*Leptosphaeria* sp.	Pleosporales	0.023		Endophytes, saprotrophs, pathogens
110.	*Leptosphaerulina australis* McAlpine	Pleosporales		0.014	Endophyte
111.	***Lophiostoma corticola* (Fuckel) E.C.Y. Liew, Aptroot & K.D. Hyde + *Lophiostoma* sp.**	Pleosporales		0.788	Pathogens
112.	***Lophodermium pinastri* (Schrad.) Chevall. + *L. seditiosum* Minter, Staley & Millar + *Lophodermium* sp.**	Rhytismatales	0.107	0.003	Pathogens
113.	*Lophotrichus* sp.	Microascales	0.017		Patogen, coprophilus, human pathogen
114.	*Macroconia sphaeriae* (Fuckel) Gräfenhan & Schroers	Hypocreales		0.013	Saprotroph, mycoparasitic
115.	*Magnohelicospora fuscospora* (Linder) R.F. Castañeda, Hern.-Restr. & Gené	Incertae sedis	0.269		Saprotroph
116.	*Massarina* sp.	Pleosporales		0.002	Saprotroph
117.	*Megacapitula villosa* J.L. Chen & Tzean	Incertae sedis	0.001		Saprotroph
118.	*Melanospora kurssanoviana* (Beliakova) Czerepan	Melanosporales	0.009		Saprotroph, mycoparasitic
119.	*Metarhizium marquandii* (Massee) Kepler, S.A. Rehner & Humber	Hypocreales	0.495		Endophyte
120.	*Meyerozyma guilliermondii* (Wick.) Kurtzman & M. Suzuki	Saccharomycetales	0.003	0.022	Coprophilous, human pathogen
121.	*Micarea adnata* Coppins	Lecanorales	0.006		Lichenicolous
122.	Microascaceae	Microascales	0.002		
123.	***Microdochium* sp.**	Amphisphaeriales	0.063	0.001	Pathogen
124.	*Microthecium fimicola* (E.C. Hansen) Y. Marín, Stchigel, Guarro & Cano + *M. quadrangulare* (Dania García, Stchigel & Guarro) Y. Marín, Stchigel, Guarro & Cano	Melanosporales	0.012	0.002	Saprotrophs
125.	*Minutisphaera parafimbriatispora* Raja, Oberlies, Shearer & A.N. Mill	Minutisphaerales	0.017		Saprotroph, aquatic
126.	*Mollisia* sp.	Helotiales	0.021		Saprotroph
127.	***Monographella nivalis* (Schaffnit) E. Müll**	Amphisphaeriales	0.004		Pathogen
128.	Montagnulaceae	Pleosporales	0.005		Saprotrophs, endophytes, pathogens
129.	*Mycofalcella calcarata* Marvanová, Om-Kalth. & J. Webster	Helotiales	0.002		Saprotroph, aquatic
130.	***Myco*** ***sphaerella tassiana* (De Not.) Johanson**	Capnodiales	0.008		Pathogen, saprotroph
131.	*Myrmecridium schulzeri* (Sacc.) Arzanlou, W. Gams & Crous	Myrmecridiales	0.010		Saprotroph
132.	*Naevala perexigua* (Roberge ex Desm.) K. Holm & L. Holm	Helotiales		0.001	Saprotroph
133.	*Nakazawaea anatomiae* (Zwillenb.) Kurtzman & Robnett + *N. populi* (Hagler, Mend.-Hagler & Phaff) Kurtzman & Robnett	Saccharomycetales	0.016	12.941	Saprotrophs
134.	***Nectria* sp.**	Hypocreales	0.032		Pathogens, saprotrophs
135.	Nectriaceae	Hypocreales	0.432		
136.	***Neoascochytaexitialis* (Morini) Qian Chen & L. Cai**	Pleosporales	0.012		Pathogen
137.	*Neobulgaria premnophila* Roll-Hansen & H. Roll-Hansen + *N. pura* (Pers.) Petr. + *Neobulgaria* sp.	Helotiales	0.684		Saprotrophs
138.	***Neocatenulostroma germanicum* (Crous & U. Braun) Quaedvl. & Crous**	Capnodiales		0.001	Pathogen
139.	*Neocucurbitaria cava* (Schulzer) Gruyter, Aveskamp & Verkley	Pleosporales		0.002	Saprotroph
140.	***Neofabraea perennans* Kienholz**	Helotiales		0.009	Pathogen
141.	***Neolepto*** ***sphaeria rubefaciens* (Togliani) Gruyter, Aveskamp & Verkley**	Pleosporales		0.003	Pathogen
142.	***Neonectria candida* (Ehrenb.) Rossman, L. Lombard & Crous + *Neonectria* sp.**	Hypocreales	0.560	0.763	Pathogen
143.	***Neopyrenochaeta acicola* ((Moug. & Lév.) Valenz.-Lopez, Crous, Stchigel, Guarro & Cano** ** + *N. inflorescentiae* (Crous, Marinc. & M.J. Wingf.) Valenz.-Lopez, Crous, Stchigel, Guarro & Cano**	Pleosporales	0.014	0.058	Pathogens, saprotrophs
144.	*Neosetophoma clematidis* Wijayaw., Camporesi & K.D. Hyde	Pleosporales		0.046	Saprotroph
145.	*Neurospora terricola* Goch. & Backus	Sordariales	0.004		Saprotroph
146.	*Niesslia mucida* (W. Gams) W. Gams & Stielow	Hypocreales	0.004		Saprotroph
147.	*Nigrograna mycophila* Jaklitsch, Friebes & Voglmayr	Pleosporales		0.007	Saprotroph, mycoparasitic
148.	***Nigro*** ***spora oryzae* (Berk. & Broome) Petch**	Incertae sedis	0.535		Saprotroph, pathogen
149.	*Ochrocladosporium elatum* (Harz) Crous & U. Braun	Pleosporales	0.022	0.084	Endophyte
150.	*Oedocephalum nayoroense* Ts. Watan	Pezizales	0.049		Saprotroph
151.	Onygenales	Onygenales	0.005		
152.	**Ophiostomataceae**	Ophiostomatales	0.790		Pathogens
153.	*Orbilia auricolor* (A. Bloxam) Sacc.	Orbiliales		0.026	Saprotroph
154.	Orbiliaceae	Orbiliales	0.006		
155.	*Pachyramichloridium pini* (de Hoog & Rahman) C. Nakash., Videira & Crous	Capnodiales	0.017		Pathogen
156.	*Papulaspora pisicola* J.F.H. Beyma	Incertae sedis	0.019		Saprotroph
157.	***Paraphoma chrysanthemicola* (Hollós) Gruyter, Aveskamp & Verkley + *P. radicina* (McAlpine) Morgan-Jones & J.F. White + *Paraphoma* sp.**	Pleosporales		4.852	Saprotrophs, pathogens
158.	*Penicillium citreonigrum* Dierckx + *P. citreosulfuratum* Biourge + *P. georgiense* S.W. Peterson & B.W. Horn + *P. glandicola* (Oudem.) Seifert & Samson + *P. halotolerans* Frisvad, Houbraken & Samson + *P. lapidosum* Raper & Fennell + *P. nothofagi* Houbraken, Frisvad & Samson + *P. raphiae* Houbraken, Frisvad & Samson + *P. roseomaculatum* Biourge + *P*. *sacculum* E. Dale + *P. unicum* Tzean, J.L. Chen & Shiu + *P. virgatum* Nirenberg & Kwaśna + *Penicillium* sp. + *Talaromyces luteus* C.R. Benj.	Eurotiales	0.295	0.001	Saprotrophs
159.	*Periconia* sp.	Pleosporales	0.012		Endophyte
160.	*Petriella sordida* (Zukal) G.L. Barron & J.C. Gilman	Microascales		0.001	Coprophilous
161.	*Phacidium lacerum* Fr. + *Phacidium* sp.	Phacidiales	0.027		Saprotroph
162.	***Phaeoacremonium cinereum* Gramaje, Mohammadi, Banihash., Armengol & L. Mostert + *P. hungaricum* Essakhi, Mugnai, Surico & Crous**	Togniniales		0.044	Pathogens
163.	***Phaeoisaria loranthacearum* Crous & R.K. Schumach. + *P. sparsa* B. Sutton**	Xylariales	0.347		Saprotrophs, coprophilous
164.	***Phaeomoniella* sp.**	Phaeomoniellales	0.001		
165.	***Phaeosphaeria* sp.**	Pleosporales	0.007		Pathogens
166.	Phaeosphaeriaceae	Pleosporales	0.013		
167.	***Phaeosphaeriopsis* sp.**	Pleosporales		0.032	Pathogens, saprotrophs
168.	*Phialocephala* sp.	Helotiales	0.004		Saprotrophs
169.	***Phialophora* sp.**	Chaetothyriales		10.291	Saprotrophs, pathogens
170.	***Phoma boeremae* Gruyter + *Phoma* sp.**	Pleosporales	0.010	0.007	Saprotrophs, pathogens
171.	***Phomopsis phaseoli* (Desm.) Sacc. + *P. velata* (Sacc.) Traverso + *Phomopsis* sp.**	Diaporthales		1.186	Pathogens, saprothrophs endophytes
172.	*Physcia tenella* (Scop.) DC.	Caliciales		0.001	Lichenicolous
173.	*Pilophorus strumaticus* Nyl. ex Cromb	Lecanorales		0.001	Lichenicolous
174.	*Plagiostoma jonesii* Senan. & K.D. Hyde	Diaporthales		0.031	Saprotroph, endophyte
175.	***Plectosphaerella cucumerina* (Lindf.) W. Gams + *P. niemeijerarum* L. Lombard**	Glomerellales	0.140	0.014	Pathogens
176.	Pleosporaceae	Pleosporales	0.003		
177.	Pleosporales	Pleosporales	0.161	0.504	
178.	*Pleotrichocladium opacum* (Corda) Hern.-Restr., R.F. Castañeda & Gené	Pleosporales	0.307	0.013	Saprotroph, aquatic
179.	*Pleurophoma ossicola* Crous, Krawczynski & H.-G. Wagner + *Pleurophoma* sp.	Xylariales	0.016	0.005	Saprotroph
180.	*Podospora appendiculata* (Auersw. ex Niessl) Niessl + *P. bulbillosa* (W. Gams & Mouch.) X. Wei Wang & Houbraken. + *P. leporina* (Cain) Cain + *Podospora* sp.	Sordariales	0.074		Saprotroph, coprophilous
181.	*Preussia flanaganii* Boylan + *P. typharum* (Sacc.) Cain	Pleosporales	0.058		Saprotrophs, endophytes, coprophilous
182.	*Pseudeurotium hygrophilum* (Sogonov, W. Gams, Summerb. & Schroers) Minnis & D.L. Lindner + *P. ovale* Stolk + *P. zonatum* J.F.H. Beyma	Thelebolales	0.804		Saprotrophs, human pathogens
183.	***Pseudocercospora angolensis* (T. Carvalho & O. Mendes) Crous & U. Braun**	Mycosphaerellales	0.004		Pathogen
184.	*Pseudogymnoascus pannorum* (Link) Minnis & D.L. Lindner + *P. roseus* Raillo	Thelebolales	0.068		Saprotrophs
185.	***Pyrenochaeta* sp.**	Incertae sedis	0.105	0.005	Pathogen, saprotroph
186.	***Pyrenochaetopsis leptospora* (Sacc. & Briard) Gruyter, Aveskamp & Verkley + *P. microspora* (Gruyter & Boerema) Gruyter, Aveskamp & Verkley**	Pleosporales	0.007	0.001	Pathogens, saprotrophs, endophytes
187.	Pyronemataceae	Pezizales	0.081		
188.	*Saccharomyces cerevisiae* (Desm.) Meyen	Saccharomycetales	0.001		Saprotroph
189.	*Schizothecium glutinans* (Cain) N. Lundq	Sordariales	0.015		Saprotroph, coprophilous
190.	*Scolecobasidium constrictum* E.V. Abbott + *S. umbrinum* (Ach.) Arnold	Incertae sedis	0.016	0.002	Saprotrophs, endophytes
191.	*Scutellinia scutellata* (L.) Lambotte	Pezizales	0.005		Saprotroph
192.	***Scytalidium lignicola* Pesante + *S. multiseptatum* Hol.-Jech**	Helotiales	0.055	0.001	Pathogens, saprotrophs, mycoparasitic
193.	Sordariales		0.008		
194.	Sordariomycetes		0.211	0.003	
195.	***Sphaeropsis sapinea* (Fr.) Dyko & B. Sutton**	Botryosphaeriales	0.003		Pathogen
196.	Sporormiaceae	Pleosporales	0.003		
197.	***Sporothrix dentifunda* Aghayeva & M.J. Wingf. + *S. stenoceras* (Robak) Z.W. de Beer, T.A. Duong & M.J. Wingf. + *S. narcissi* (Limber) Z.W. de Beer, T.A. Duong & M.J. Wingf**	Ophiostomatales	0.161	0.001	Pathogens, saprotrophs
198.	***Stemphylium herbarum* E.G. Simmons + *S. majusculum* E.G. Simmons + *S. vesicarium* (Wallr.) E.G. Simmons**	Pleosporales	0.027		Pathogens
199.	*Subramaniula flavipila* X. Wei Wang & Samson	Sordariales	0.014		Saprotroph
200.	*Sydowia polyspora* (Bref. & Tavel) E. Müll	Dothideales	0.004	1.028	Pathogen, endophyte, saprotroph
201.	*Tetracladium furcatum* Descals + *T. setigerum* (Grove) Ingold + *Tetracladium* sp.	Helotiales	1.171	0.862	Saprotrophs
202.	***Thelonectria blackeriella* + *T. olida* (Wollenw.) Wollenw. + *T. nodosa* Salgado & P. Chaverri**	Hypocreales	0.012	0.006	Pathogens
203.	*Tricharina* sp.	Pezizales	1.55		Saprotrophs
204.	*Trichocladium asperum* Harz + *T. griseum* (Traaen) X. Wei Wang & Houbraken	Sordariales	0.593		Saprotrophs
205.	*Trichoderma aerugineum* Jaklitsch + *T. hamatum* (Bonord.) Bainier + *T. koningiopsis* Samuels, Carm. Suárez & H.C. Evans + *T. martiale* Samuels + *T. neokoningii* Samuels & Soberanis + *T. piluliferum* J. Webster & Rifai + *T. Polysporum* (Link) Rifai + *T. pubescens* Bissett + *T. stilbohypoxyli* Samuels & Schroers + *T. viride* Pers. + *Trichoderma* sp.	Hypocreales	19.464	0.001	Saprotrophs
206.	*Tricladium splendens* Ingold	Helotiales	0.040	0.057	Saprotroph, acquatic
207.	***Truncatella an gustata* (Pers.) S. Hughes + *T. restionacearum* S.J. Lee & Crous**	Amphisphaeriales	0.003	0.001	Pathogens
208.	***Valsa malicola* Z. Urb. + *V. sordida* Sacc. + *V. leucostoma* (Pers.) Fr.**	Diaporthales	0.012	0.214	Pathogens
209.	Valsaceae	Diaporthales	0.003		
210.	*Venturia hystrioides* (Dugan, R.G. Roberts & Hanlin) Crous & U. Braun	Venturiales	0.018		Pathogen
211.	Venturiaceae sp.	Venturiales		0.001	
212.	***Verticillium dahliae* Kleb. + *V. longisporum* (C. Stark) Karapapa, Bainbr. & Heale**	Glomerellales	0.029		Pathogens, saprotrophs
213.	***Volutella ciliata* (Alb. & Schwein.) Fr. + *Volutella* sp.**	Hypocreales	0.009	0.009	Saprotrophs, pathogen
214.	*Xanthoparmelia subchalybaeizans* (Hale) G. Amo, A. Crespo, Elix & Lumbsch	Lecanorales	0.005		Lichenicolous
215.	*Xenochalara* sp.	Helotiales	0.033		Saprotroph
216.	*Xenopolyscytalum pinea* Crous + *Xenopolyscytalum* sp.	Helotiales	0.001	0.001	Saprotrophs
217.	***Xenoramularia arxii* Videira, Crous & U. Braun**	Capnodiales		0.001	Pathogen
218.	Xylariales	Xylariales	0.061		
219.	*Yamadazyma mexicana* (M. Miranda, Holzschu, Phaff & Starmer) Billon-Grand	Saccharomycetales		0.039	Saprotroph
220.	*Yarrowia lipolytica* (Wick., Kurtzman & Herman) Van der Walt & Arx	Saccharomycetales	0.001		Saprotroph
221.	*Zalerion* sp.	Lulworthiales		0.001	Saprotroph, aquatic
222.	*Zopfiella marina* Furuya & Udagawa + *Z. pilifera* Udagawa & Furuya	Sordariales	0.027		Saprotrophs, aquatic
**Frequency of Ascomycota**			**45.299**	**68.697**	
**Number of taxa Ascomycota**			263	178	
**Basidiomycota**					
1.	***Aecidium* sp.**	Pucciniales	0.034		Pathogen
2.	Agaricales		0.054		
3.	Agaricomycetes		0.008	0.074	
4.	Agaricostilbomycetes		0.001		
5.	*Apiotrichum dulcitum* (Berkhout) Yurkov & Boekhout + *A. gracile* (Weigmann & A. Wolff) Yurkov & Boekhout	Trichosporonales	0.047		Saprotrophs
6.	***Armillaria mellea* (Vahl) P. Kumm**	Agaricales	0.025		Pathogen
7.	*Athelia acrospora* Jülich	Atheliales		0.001	Saprotroph
8.	Atheliaceae	Atheliales	0.023		
9.	***Aurantiporus fissilis* (Berk. & M.A. Curtis) H. Jahn ex Ryvarden**	Polyporales		0.002	Saprotroph, pathogen
10.	Auriculariales		0.004		
11.	Basidiomycota		0.031	0.038	
12.	*Bensingtonia* sp.	Agaricostilbales	0.001		Saprotroph
13.	*Bjerkandera adusta* (Willd.) P. Karst	Polyporales		0.002	Saprotroph, pathogen
14.	*Buckleyzyma aurantiaca* (Saito) Q.M. Wang, F.Y. Bai, M. Groenew. & Boekhout	Buckleyzymales	0.048	0.007	Saprotroph
15.	*Bullera crocea* Buhagiar	Tremellales	0.008	0.001	Saprotroph
16.	*Bulleromyces albus* Boekhout & Á. Fonseca	Tremellales	0.001	0.001	Saprotroph
17.	*Burgoa anomala* (Hotson) Goid	Cantharellales		0.009	Saprotroph
18.	*Camarophyllus* sp.	Agaricales	0.001		Mycorrhizal
19.	Cantharellales		0.002		
20.	***Chondrostereum purpureum* (Pers.) Pouzar**	Agaricales		0.018	Pathogen, saprotroph
21.	*Coprinellus disseminatus* (Pers.) J.E. Lange	Agaricales		0.230	Saprotroph
22.	*Cryptococcus tephrensis* Vishniac + *Cryptococcus* sp.	Tremellales	0.220	0.406	Saprotrophs, endophytes
23.	*Curvibasidium pallidicorallinum* Golubev, Fell & N.W. Golubev	Incertae sedis		0.001	Mycocinogenic
24.	Cystobasidiomycetes		0.003		
25.	*Cystobasidium pinicola* (F.Y. Bai, L.D. Guo & J.H. Zhao) Yurkov, Kachalkin, H.M. Daniel, M. Groenew., Libkind, V. de Garcia, Zalar, Gouliam., Boekhout & Begerow + *C. psychroaquaticum* A.M. Yurkov, Kachalkin, H.M. Daniel, M. Groenew., Libkind, V. de Garcia, Zalar, Gouliamova, Boekhout & Begerow	Cystobasidiales	0.002	0.016	Saprotrophs, mycoparasitic
26.	Cystofilobasidiales	Cystofilobasidiales	0.004	0.001	
27.	*Cystofilobasidium infirmominiatum* (Fell, I.L. Hunter & Tallman) Hamam., Sugiy. & Komag. + *C. macerans* J.P. Samp.	Cystofilobasidiales	0.012	0.001	Saprotrophs, acquatic
28.	*Daedaleopsis confragosa* (Bolton) J. Schröt	Polyporales		0.001	Saprotroph
29.	*Efibulobasidium* sp.	Sebacinales	0.020		Mycorrhizal
30.	***Entyloma gaillardianum* Vánky + *E. polysporum* (Peck) Farl.**	Entylomatales	0.044		Pathogens
31.	Erythrobasidiales	Erythrobasidiales	0.001	0.001	
32.	*Erythrobasidium hasegawae* (Y. Yamada & Komag.) Hamam., Sugiy. & Komag	Erythrobasidiales		0.008	Saprotroph
33.	*Exidiopsis* sp.	Auriculariales		0.001	Saprotroph
34.	***Exobasidium arescens* Nannf. + *Exobasidium* sp.**	Exobasidiales	0.001	0.001	Pathogen
35.	*Fellomyces* sp.	Tremellales		0.001	Saprotroph
36.	*Fellozyma inositophila* (Nakase & M. Suzuki) Q.M. Wang, F.Y. Bai, M. Groenew. & Boekhout	Incertae sedis	0.007		Saprotroph
37.	*Fibulobasidium inconspicuum* Bandoni	Tremellales	0.004	0.379	Saprotroph
38.	*Filobasidium wieringae* (Á. Fonseca, Scorzetti & Fell) Xin Zhan Liu, F.Y. Bai, M. Groenew. & Boekhout	Filobasidiales	0.008		Saprotroph
39.	***Fomitopsis pinicola* (Sw.) P. Karst**	Polyporales		0.005	Pathogen, saprotroph
40.	*Geotrichopsis mycoparasitica* Tzean & Estey	Incertae sedis	0.033		Mycoparasitic
41.	*Gymnopus androsaceus* (L.) Della Magg. & Trassin	Agaricales	0.001		Saprotroph, mycoparasitic
42.	*Hannaella zeae* (O. Molnár & Prillinger) F.Y. Bai & Q.M. Wang	Tremellales	0.047		Saprotroph, endophyte
43.	*Hebeloma mesophaeum* (Pers.) Quél	Agaricales	0.007		Mycorrhizal
44.	Hydnaceae	Cantharellales	0.004		
45.	Hygrophoraceae	Agaricales	0.008		
46.	*Hymenogaster arenarius* Tul. & C. Tul.	Agaricales	0.005		Ectomycorrhizal
47.	*Hyphodontia pallidula* (Bres.) J. Erikss	Hymenochaetales	0.003		Saprotroph
48.	*Hypochnicium lundellii* (Bourdot) J. Erikss	Polyporales	0.012		Saprotroph
49.	*Inocybe curvipes* P. Karst	Agaricales	0.043		Ectomycorrhizal
50.	***Itersonilia perplexans*** **Derx**	Cystofilobasidiales	0.001		Pathogen
51.	*Kockovaella machilophila* Cañ.-Gib., M. Takash., Sugita & Nakase	Tremellales	0.001		
52.	*Kondoa yuccicola* (Nakase & M. Suzuki) Q.M. Wang, M. Groenew., F.Y. Bai & Boekhout	Agaricostilbales		0.012	Saprotroph
53.	*Kwoniella newhampshirensis* K. Sylvester, Q.M. Wang & Hittinger + *K. pini* (Golubev & I. Pfeiff.) Xin Zhan Liu, F.Y. Bai, M. Groenew. & Boekhout	Tremellales	0.016	0.003	Entomopathogenic
54.	*Laccaria* sp.	Agaricales		0.001	Ectomycorrhizal
55.	*Lachnella alboviolascens* (Alb. & Schwein.) Fr.	Agaricales		0.007	Saprotroph
56.	*Leptosporomyces galzinii* (Bourdot) Jülich	Atheliales	0.054		Saprotroph
57.	Leucosporidiales	Leucosporidiales	0.007		
58.	*Malassezia globosa* Midgley, E. Guého & J. Guillot + *M. restricta* E. Guého, J. Guillot & Midgley +	Malasseziales	0.016	0.001	Human pathogens
59.	*Marasmius cohaerens* (Pers.) Cooke & Quél	Agaricales	0.008		Saprotroph
60.	Microbotryomycetes		0.042		
61.	*Minimedusa polyspora* (Hotson) Weresub & P.M. LeClair	Cantharellales	0.069		Saprotroph, mycoparasitic
62.	*Mrakia frigida* (Fell, Statzell, I.L. Hunter & Phaff) Y. Yamada & Komag. + *Mrakia* sp.	Cystofilobasidiales	0.012	0.001	Saprotroph
63.	*Mycena aurantiomarginata* (Fr.) Quél. + *M. galericulata* (Scop.) Gray	Agaricales	0.003	0.001	Saprotroph
64.	*Naganishia cerealis* (Passoth, A.-C. Andersson, Olstorpe, Theelen, Boekhout & Schnürer) Xin Zhan Liu, F.Y. Bai, M. Groenew. & Boekhout + *N. diffluens* (Zach) Xin Zhan Liu, F.Y. Bai, M. Groenew. & Boekhout	Tremellales	0.021	0.001	Saprotroph
65.	*Oberwinklerozyma silvestris* Golubev & Scorzetti ex Q.M. Wang, F.Y. Bai, M. Groenew. & Boekhout	Incertae sedis	0.012		
66.	*Oliveonia* sp.	Auriculariales		0.008	Saprotroph
67.	***Peniophora* sp.**	Russulales		0.593	Pathogen, saprotroph
68.	*Phaeotremella frondosa* (Fr.) Spirin & V. Malysheva + *P. roseotincta* (Lloyd) V. Malysheva	Tremellales	0.001	0.123	Saprotrophs, mycoparasites
69.	*Phloeomana speirea* (Fr.) Redhead	Agaricales		0.024	Saprotroph, aquatic
70.	*Piskurozyma* sp.	Filobasidiales	0.024		Saprotroph
71.	*Psathyrella squamosa* (P. Karst.) A.H. Sm.	Agaricales	0.004		Saprotroph
72.	*Rhodotorula glutinis* (Fresen.) F.C. Harrison + *Rhodotorula* sp.	Sporidiobolales	0.003	0.001	Saprotrophs
73.	*Saitozyma podzolica* (Babeva & Reshetova) Xin Zhan Liu, F.Y. Bai, M. Groenew. & Boekhout	Tremellales		0.001	Saprotroph
74.	*Sakaguchia lamellibrachiae* (Nagah., Hamam., Nakase & Horikoshi) Q.M. Wang, F.Y. Bai, M. Groenew. & Boekhout	Sakaguchiales		0.027	Saprotroph
75.	Sebacinales	Sebacinales	0.392	0.001	
76.	*Serendipita vermifera* Oberw	Sebacinales		0.017	Endophyte, mycorrhizal
77.	***Serpula himantioides* (Fr.) P. Karst**	Boletales	0.001		Saprotroph, pathogen
78.	*Sirotrema translucens* (H.D. Gordon) Bandoni	Tremellales		0.001	Saprotroph
79.	*Sistotremastrum* sp.	Trechisporales		0.001	Saprotroph
80.	*Slooffia pilatii* (F.H. Jacob, Faure-Reayn. & Berton) Q.M. Wang, F.Y. Bai, M. Groenew. & Boekhout	Incertae sedis		0.001	Saprotroph
81.	*Solicoccozyma fuscescens* (Golubev) Yurkov + *S. phenolica* (Á. Fonseca, Scorzetti & Fell) A.M. Yurkov + *S. terrea* (Di Menna) A.M. Yurkov + *S. terricola* (T.A. Pedersen) Yurkov	Filobasidiales	2.451	0.004	Saprotrophs
82.	***Sporobolomyces roseus* Kluyver & C.B. Niel + *Sporobolomyces* sp.**		0.008	0.001	
83.	*Stilbum* sp.	Agaricostilbales		0.018	Saprotroph
84.	*Symmetrospora coprosmae* (Hamam. & Nakase) Q.M. Wang, F.Y. Bai, M. Groenew. & Boekhout + *S. gracilis* (Derx) Q.M. Wang, F.Y. Bai, M. Groenew. & Boekhout	Incertae sedis	0.005	0.001	Saprotrophs
85.	*Tausonia pullulans* (Lindner) Xin Zhan Liu, F.Y. Bai, J.Z. Groenew. & Boekhout	Cystofilobasidiales	0.094	0.012	Saprotrophs
86.	Thelephoraceae	Thelephorales	0.058		Pathogens
87.	*Tomentella* sp.	Thelephorales		0.001	Ectomycorrhizal
88.	*Tremella encephala* Pers.	Tremellales		0.003	Saprotroph
89.	Tremellales		0.014	0.001	Saprotrophs
90.	Tremellomycetes		0.003		
91.	Tricholomataceae	Agaricales	0.004		
92.	*Trichosporon otae* Sugita, Takshima & Kikuchi	Trichosporonales	0.003		Human pathogen
93.	Tulasnellaceae	Cantharellales		0.005	
94.	*Typhula incarnata* Lasch	Agaricales	0.004		Pathogen
95.	*Pappia fissilis* (Berk. & M.A. Curtis) Zmitr	Polyporales	0.004		Saprotroph
96.	*Vishniacozyma carnescens* (Verona & Luchetti) Xin Zhan Liu, F.Y. Bai, M. Groenew. & Boekhout + *V. globispora* (B.N. Johri & Bandoni) Xin Zhan Liu, F.Y. Bai, M. Groenew. & Boekhout + *V. victoriae* (M.J. Montes, Belloch, Galiana, M.D. García, C. Andrés, S. Ferrer, Torr.-Rodr. & J. Guinea) Xin Zhan Liu, F.Y. Bai, M. Groenew. & Boekhout	Tremellales	0.007	0.005	Pathogens, saprotrophs
**Frequency Basidiomycota**			4.119	2.076	
**Number of Basidiomycota taxa**			81	59	
			**Frequency**		
Oomycota			1.199	0.002	
**Culturable fungi**			53.062	70.780	
**Non-culturable fungi**			25.645	17.435	
**Other Kingdoms**			15.822	11.728	
**No sequence in NCBI database**			4.272	0.055	
			**Number**		
Total OTUs			69,467 ^a^	70,218 ^a^	
Culturable fungal OTUs			44,506 ^a^	53,592 ^a^	
Taxa			474 ^a^	309 ^a^	
Fungal taxa			364 ^a^	242 ^a^	
Margalef’s diversity index–*DMg*			65.54	21.72	
Shannon’s diveristy index–*H*			2.55	0.77	
Simpson’s diversity index–*D*			0.21	0.74	
Shannon’s evenness index–*E*			0.39	0.17	
Berger-Parker’s dominance index–*d*			0.20	0.46	
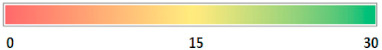					

Percentage of variation. Pathogens are in bold. ^a^ Indicates a statistically significant difference according to a *χ*^2^-test, *p* < 0.001.

## Data Availability

Data supporting reported results can be found at https://figshare.com/s/2c89719675a6859ee8a6 (accessed on 11 April 2021).
